# BCNNM: A Framework for *in silico* Neural Tissue Development Modeling

**DOI:** 10.3389/fncom.2020.588224

**Published:** 2021-01-20

**Authors:** Dmitrii V. Bozhko, Georgii K. Galumov, Aleksandr I. Polovian, Sofiia M. Kolchanova, Vladislav O. Myrov, Viktoriia A. Stelmakh, Helgi B. Schiöth

**Affiliations:** ^1^JetBrains Research Department, Space Office Center, Saint Petersburg, Russia; ^2^Department of Biology, University of Puerto Rico at Mayaguez, Mayaguez, PR, United States; ^3^Theodosius Dobzhansky Center for Genome Bioinformatics, St. Petersburg State University, Saint Petersburg, Russia; ^4^Neuroscience Center, Helsinki Institute of Life Science, University of Helsinki, Helsinki, Finland; ^5^Department of Neuroscience and Biomedical Engineering, Aalto University, Espoo, Finland; ^6^Skolkovo Institute of Science and Technology, Center of Life Sciences, Moscow, Russia; ^7^Department of Neuroscience, Functional Pharmacology, Uppsala University, Uppsala, Sweden; ^8^Institute for Translational Medicine and Biotechnology, Sechenov First Moscow State Medical University, Moscow, Russia

**Keywords:** brain organoid, tissue development, neurogenesis, simulation, axon guidance, neuronal connectivity

## Abstract

Cerebral (“brain”) organoids are high-fidelity *in vitro* cellular models of the developing brain, which makes them one of the go-to methods to study isolated processes of tissue organization and its electrophysiological properties, allowing to collect invaluable data for *in silico* modeling neurodevelopmental processes. Complex computer models of biological systems supplement *in vivo* and *in vitro* experimentation and allow researchers to look at things that no laboratory study has access to, due to either technological or ethical limitations. In this paper, we present the Biological Cellular Neural Network Modeling (BCNNM) framework designed for building dynamic spatial models of neural tissue organization and basic stimulus dynamics. The BCNNM uses a convenient predicate description of sequences of biochemical reactions and can be used to run complex models of multi-layer neural network formation from a single initial stem cell. It involves processes such as proliferation of precursor cells and their differentiation into mature cell types, cell migration, axon and dendritic tree formation, axon pathfinding and synaptogenesis. The experiment described in this article demonstrates a creation of an *in silico* cerebral organoid-like structure, constituted of up to 1 million cells, which differentiate and self-organize into an interconnected system with four layers, where the spatial arrangement of layers and cells are consistent with the values of analogous parameters obtained from research on living tissues. Our *in silico* organoid contains axons and millions of synapses within and between the layers, and it comprises neurons with high density of connections (more than 10). In sum, the BCNNM is an easy-to-use and powerful framework for simulations of neural tissue development that provides a convenient way to design a variety of tractable *in silico* experiments.

## 1. Introduction

Acquiring more precise knowledge about the molecular processes that occur in neural tissue is crucial for understanding the mechanisms involved in normal nervous system development and progression of neurodevelopmental disorders, effects of exposure to harmful chemicals or infections during embryonic and fetal stages on the nervous system, as well as post-traumatic tissue recovery (Amunts et al., 2013; John H. Byrne, 2014). Such insights will also be highly advantageous for compound screening in search for new drug candidates at early stages of testing (Xu et al., 2016; Watanabe et al., 2017; Zhou et al., 2017). Certain human tissues, like those in the brain or in the spinal cord, cannot be readily accessed experimentally, which is why their development, normal functioning and disease remain understudied in some aspects (Arlotta and Pasca, 2019). Although brain organoids partially solve this problem by mimicking many key features of early human brain development at the molecular, cellular, structural and functional levels, some events such as the formation of distinct cortical neuronal layers and gyrification, are not fully recapitulated; the fidelity of circuit formation and maturation in organoids remains unclear (Andrews and Nowakowski, 2019; Qian et al., 2019). Studies of many disease-related processes are currently problematic as they require evaluation of the aspects of neuronal maturation that are not well-represented in organoids (Andrews and Nowakowski, 2019). In addition, none of the *in vivo* or *in vitro* methods allow researchers to keep a record of all the parameters of interest within the studied system throughout the entire experimental timeframe. There is increasing incentive to perform such experiments with computer simulations, wherein every singular event can be registered and analyzed.

Numerous simulations in computational neuroscience focus on different features and organization levels of developing systems. Existing models of one category describe various individual aspects of cell functioning, for example the effects of gene expression on regional specification in the nervous system (Giacomantonio and Goodhill, 2014), neuronal migration and polarization (Caffrey et al., 2014), axon and dendrite growth, guidance and branching (Padmanabhan and Goodhill, 2018). Such software allows to model gene regulatory networks underlying specific developmental processes and to evaluate the plausibility of different networks, assess the role of secreted factors and extracellular matrix on neuronal migration and positioning and delve into many other complex aspects of neurodevelopment (Caffrey et al., 2014; Giacomantonio and Goodhill, 2014). In some cases these models can serve as starting points to incorporate additional molecular interactions and pathways and to identify new regulatory mechanisms (Padmanabhan and Goodhill, 2018). However, despite dealing with specific details of neural development, these models do not paint a holistic picture (Goodhill, 2018) and only allow for somewhat limited freedom in analyses of interactions between various mechanisms (Giacomantonio and Goodhill, 2014); problems with model scaling and extrapolation of the results might also arise in many cases.

Models of another kind take a more generalized approach: they use complex mathematical equations and data-driven parameters to simulate events like acquisition of neocortical neurons (Gohlke et al., 2007), adult neurogenesis (Ziebell et al., 2014; Beccari et al., 2017), connectome establishment (Borisyuk et al., 2011), and interactions within it (Razetti et al., 2018). Such models represent all cells within a tissue as a unified system and do not concern with individual cells or compartments. They allow researchers to explore important questions like how parameter changes of individual cellular processes during neurogenesis affect neocortical expansion, how to describe quantitatively the effects of altered stem cell dynamic characteristics on cell counts (Gohlke et al., 2007; Ziebell et al., 2014), grow networks with realistic connectivity (Borisyuk et al., 2011) or infer principles that underlie the growth and functioning of large populations of axons (Razetti et al., 2018). These models, however, usually do not track the molecular-level events happening within and in the vicinity of individual cells, some of them are not accounting for any feedback mechanisms or for spatial components (Ziebell et al., 2014), others rely on a limited set of external influencing factors (e.g., just the mechanical factors) (Razetti et al., 2018).

Approaches that lie somewhere in the middle also exist, allowing to couple things like mechanical effects in growing tissues with molecular and genetic interactions (Zubler and Douglas, 2009; Delile et al., 2017). Such models offer practical computational frameworks to test the validity of hypotheses about developmental and morphogenetic processes both at the molecular and at the cellular level of organization. However, these are limited to several thousands or even hundreds of cells and their configuration possibilities are somewhat restricted—customization work must often be done by editing the source code directly (Zubler and Douglas, 2009; Delile et al., 2017).

We have created a framework that incorporates some of the useful features missing from the models mentioned earlier, including the user's ability to follow individual cells, register changes of a large number of different parameters, as well as to describe the cell population behavior as a whole. In this version we omitted modeling detailed mechanical interactions in favor of performance and effectiveness. Based on the kinds of experiments we planned to perform, we made an assumption that this simplification would not have a dramatic detrimental effect on the predictions of our simulations and their congruence with reality. The BCNNM framework is implemented with the principles of dynamic modeling with the possibility of probabilistic processes description and is based on the tissue level of approximation. Thanks to the description scaling of the mechanisms embedded in the model, comprehensive characterization of biological mechanisms is possible and users can choose the desired detailing level for the described processes (from shifts in intracellular enzyme concentrations to interactions of cell groups, forming supracellular structures). Spatial geometry embedded in the model makes it possible to build structures with sparse and specific connections, models of signal conduction within such structures, and to reveal peculiarities of their work.

Modeling approaches in the field of computational neuroscience take various neuronal tissue characteristics as their primary model parameters: morphological (focused on spatial tissue parameters and detailed cell descriptions) (Donohue and Ascoli, 2011; Parekh and Ascoli, 2013), biophysical (focused on neurons' electrical activity with the use of precise electrophysiological data) (Gerkin and Castro, 2015), or biochemical (focused on near-field cellular interactions) (Zubler and Douglas, 2009). Most cell- and tissue-level processes, such as cell division (Gauthier and Pohl, 2011; Tyson and Novak, 2015), tissue development (Deppmann et al., 2008) or intercellular signaling (including synaptic signaling itself) (Kepseu and Woafo, 2006), can be effectively described up to some extent in terms of biochemical reactions alone, which was the reason behind our decision to favor the biochemical approach over other possible alternatives in this work. We are aiming to provide a framework capable of modeling individual neurons, neural tissue development from a limited number of progenitor cells dividing and differentiating into many types of specialized cells, axonal patterning and guidance, synaptogenesis, post-traumatic structural and functional regeneration, as well as other cell- and tissue-level events, and having the potential to simulate *in vitro* experiments (Bernardino et al., 2008; Acimovic et al., 2011; Chen et al., 2013; Pasca, 2018; Reardon, 2018), that are looking into one or several of these complex biological processes.

## 2. Materials and Methods

This section describes the principles of modeling which became the basis for the framework, as well as specific features of the implementation of various mechanisms. The BCNNM framework is a simulation framework created using dynamical modeling methods and based on biochemical interactions. Its main constituents are:

- The core ensuring the consistency and accuracy of built-in calculations;
- Logical components describing the space and physics of biochemical signal propagation;
- Logical components describing the basic mechanisms of object creation, movement and deletion;
- Processes characterized through systems of functions and the configuration system. This allows us to carry into effect and incorporate new processes as well as alternative implementations of the existing ones.

### 2.1. Discrete Event System

The BCNNM framework is based on the Discrete Event System (DES) principle. DES approach (Cassandras and Lafortune, 2008) allows to reduce the complexity both in the model definition and in computations; it also lets us abstract from the continuous nature of real events and consider solely the keystone occurrences in the system under examination while operating with discrete sequences of actions **(Equation 1)** in time (Robinson, 2004). The specific feature of the model events is that they can occur dependently as well as independently of others. Each event contains information about its execution priority used by the state transition function.

(1) $$\begin{array}{r} E\equiv \left\{\ldots ,{\left\{\epsilon_{0},\epsilon_{1},\ldots \;\right\}}_{t-1},{\left\{\epsilon_{0},\epsilon_{1},\ldots \;\right\}}_{t},{\left\{\epsilon_{0},\epsilon_{1},\ldots \;\right\}}_{t+1},\ldots \right\} \end{array}$$

where the chronologically sorted sequence of events is denoted as *E* and ϵ is an atomic event.

Events in the model always have owners (**Equation 2**). Such an owner is a functional logical object *o*.

(2) $$\begin{array}{r} O\equiv \left\{o_{0},o_{1},\ldots ,o_{k}\right\} \end{array}$$

where all functional logical objects are forming a set *O*.

A logical object in the model is an element of abstraction, intended to unify the descriptions of components and interactions. Logical objects of the model include implementation of all possible cell compartments described by the configuration as sets of possible events combined into signaling pathways, as well as emitted and received factors. In addition, logical objects include spatial marks—a special model entity that does not have size and is capable of diffusing extracellular chemical signals. Basic logical object types are implemented as an immutable part of the BCNNM framework. The user can specify logical object descriptions based on the basic types specified in the model configuration.

Events carry information concerning which function should be executed for a particular object instance. These functions are also referred to as **mechanisms** (**Equation 3**). Their implementation is embedded in our framework: users can not introduce any changes into the core realization of mechanisms.

(3) $$\begin{array}{r} M\equiv \left\{m_{0},m_{1},\ldots ,m_{n}\right\} \end{array}$$

Basically, a mechanism is an abstract function and it can't be applied to any object without proper parametrization. A parametrized mechanism is called a **signaling pathway**. A signaling pathway in the model is a mapping of a mechanism applicable to particular types of objects specified by the user.

Configuration *B* represents entity relations between framework core principles and high-level implementation of mechanisms and objects specified by the user 
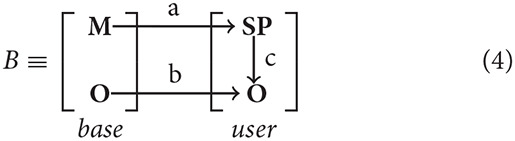


where

*a* - mapping of basic mechanisms on user-defined signaling pathways;
*b* - mapping of basic logical objects on user-defined logical objects;
*c* - mapping of user-defined signaling pathways on user-defined logical objects.

**Figure 1:**
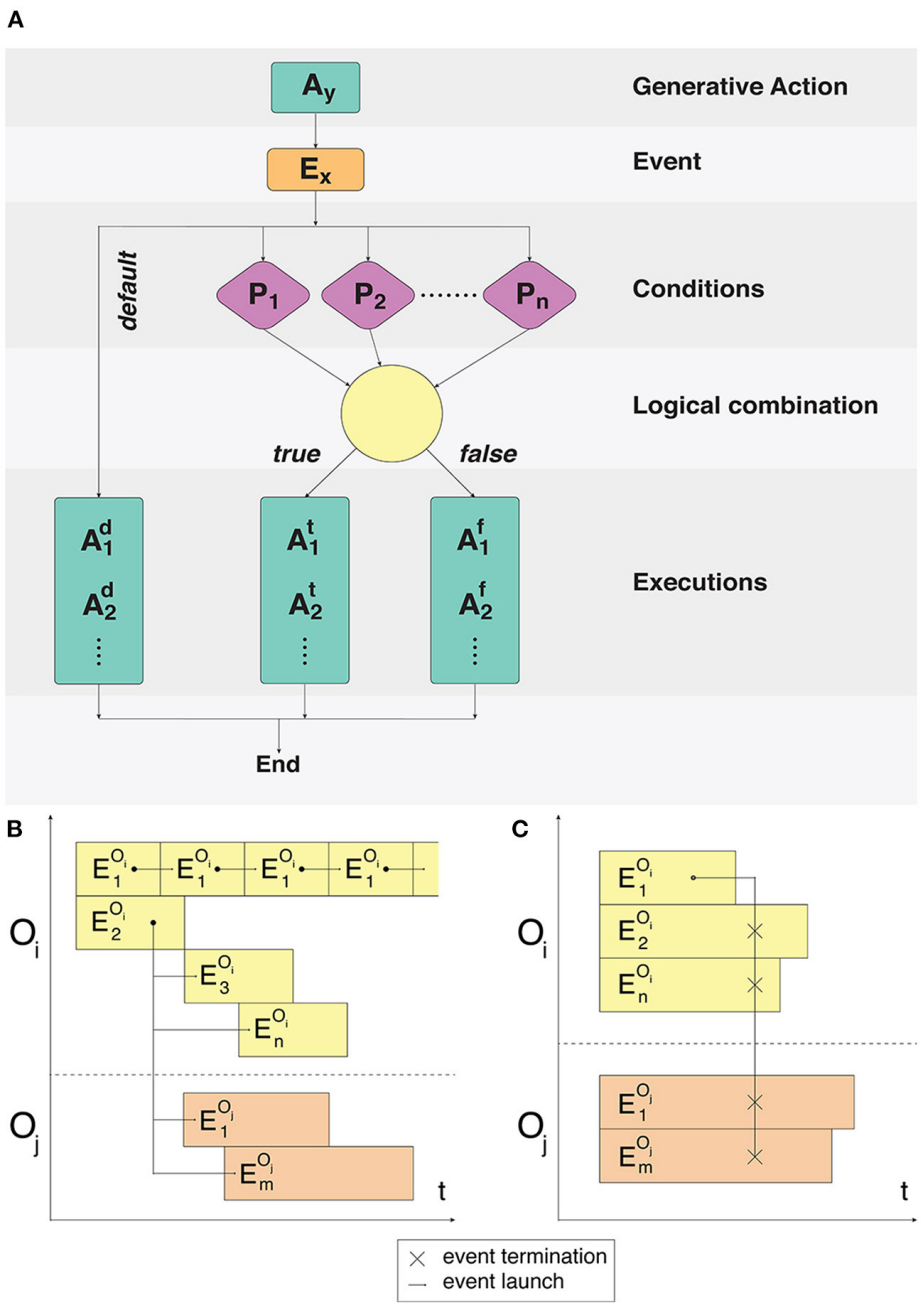
Fulfillment of event conditions and model event notation description. **(A)** An action *A*_*y*_ creates an event *E*_*x*_. The following process is split into two branches: processing of unconditional user-defined actions and a condition-based set of actions. For the condition-based branch *P*_1_ … *P*_*n*_ predicate conditions are checked during the *E*_*x*_ event processing. Conditions are combined with the predicate using logical operators *AND*/*OR*. Execution or non-execution of the predicate defines a set of events that alter the state of the system. **(B)** Cyclical execution of the $E_{1}^{O_{i}}$ event. The event $E_{2}^{O_{i}}$ triggers several subsequent events in the objects *O*_*i*_, *O*_*j*_. **(C)** Disruptive effect of the single $E_{1}^{O_{i}}$ event on all the events of the *O*_*i*_ and *O*_*j*_ objects.

Processes associated with each logical object occur continuously, causing modifications of its state, spatial position and activity. To determine any action that a logical object can perform, the concept of a signaling pathway *sp*_*i*_ ∈ *SP* is introduced, where *SP* is the set of all signaling pathways available in the system (O'Connor and Adams, 2010). Each *sp*_*i*_ launched by the Events dispatcher results in the logical object performing an action described in our framework. For each cell compartment type, a set of signaling pathways *SP*_*ct*_ ∈ *SP* is specified, which determines what kinds of actions this cell compartment can potentially perform and what conditions need to be fulfilled for each of these *SP*_*ct*_ (Figure 1A).

Depending on all the constituent space and internal state variables, certain signaling pathways can be switched on and off in the modeling process, however their total composition will never exceed the set *SP*_*ct*_ for a given compartment type. Each particular event can be configured using a set of conditions, which must be fulfilled in the cases of standard launch or interruption. Examples of event executions in a time interval are shown in Figures 1B,C.

A model individual represents a set of logical objects distributed in space. The individuals state is determined by the execution of signaling pathways of every logical object from the set of events at any given time point. The model stores an independent state of the system Ω_*t*_ at the beginning *t* of each event ϵ, and afterwards the state transition function Π synchronizes calculations from many different processes that took place simultaneously.

The initial state of the system is

(5) $$\begin{array}{r} \Omega_{0}\equiv \left[\Upsilon ,{\text{Ψ}}_{0},B\right] \end{array}$$

where

Υ is the Model, Ψ_0_ is the spatial configuration provided by the user, *B* is the biological configuration provided by the user.

The model Υ and biological *B* configurations do not change during the simulation process. Configurations of objects and their positions in space Ψ change throughout the events' processing, whereas parameters like geometry and space metrics can not be altered in the course of the simulation.

State transition function Π enables the transition from the current state Ω_*t*_ of the system to a future state Ω_*t*+1_ using the set of events *E*_*t*_ on the timestamp *t*.

(6) $$\begin{array}{r} \Omega_{t+1}\equiv \Pi \left(\Omega_{t},E_{t}\right);\;E_{t}\equiv {\left\{\epsilon_{0},\epsilon_{1},\ldots \;\right\}}_{t} \end{array}$$

The state transition function executes independent events first. Interdependent events can trigger conflicting executions that need to be resolved. Resolution of a particular conflict is based on the order of priority of the events involved.

Our system does not provide calculations for chemical reactions, however, the results of such calculations can be represented in terms of dependencies between events and conditions. This algorithm shows how a new state of the model is calculated. The *mechanismExecution* and *mechanismCondition* parameters are set by the user in the configuration for a specific simulation. Calculations derived from third-party models can be used as *mechanismCondition* as well as *mechanismPayload*. See Algorithm 1 for details.

**Algorithm 1:**
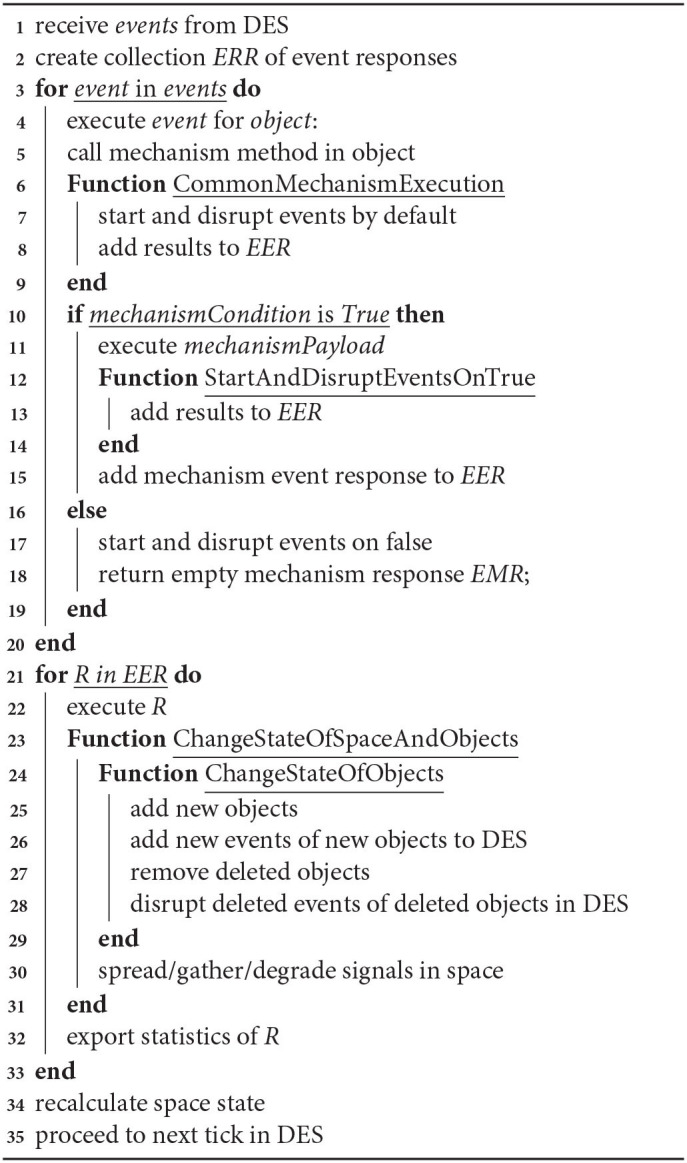
General algorithm for interaction of events. This is a general algorithm describing how events proceed and interact with each other. Algorithm calculates a new model state and updates the model state.

### 2.2. Spatial and Temporal Dimensions

Objects in the BCNNM framework can be either mobile or stationary. Based on this property, some of them can be in contact with other objects while moving, thereby changing their own position and that of others. Some objects do not have the ability to move independently, but they can also touch other objects and be displaced. All the remaining objects do not have the independent movement ability and never change their position in space, thus becoming impassable obstacles for moving objects. The model time concept is central to the simulation within our framework. The model time *T*_*m*_ is discrete with a fixed step size *t*_*m*_. Correspondence of the model with the real time can be calculated based on the actual duration of the shortest simulated process. This approach allows to simulate processes with the required level of precision.

Model space represents a simplified abstraction of the reality. To facilitate and accelerate calculations, the system space is represented as a three-dimensional integer grid *L*, with equal edges of finite length *e*_*x*_ = *e*_*y*_ = *e*_*z*_. The minimum displacement in *L* is the displacement *l*_1_. When it is applied to the coordinate *C*_0_ = (*x*_0_, *y*_0_, *z*_0_) the resulting coordinate *C*_*n*_ = (*x*_*n*_, *y*_*n*_, *z*_*n*_) is such that the Chebyshev distance between *C*_0_ and *C*_*n*_ is equal to 1 (Supplementary Figure 1). In a three-dimensional grid for the coordinate *C*_0_ the maximum possible number of neighbor coordinates with displacement *l*_1_ is 26. A multitude of such coordinates is called a neighborhood *N*_*c*_ of the coordinate *C*_0_ with radius 1. Objects in the space either occupy a single coordinate, or a sequence of coordinates (for example, the body of a neuron occupies just one coordinate, while its axon is represented as a sequence beginning with the coordinate of the cell body). Coordinate occupied by a cell is considered to be unavailable and cannot be simultaneously used by another cell. Coordinates occupied by special spatial marks or parts of cells are considered vacant and available for use by other cells. In the mechanics of the model space, such object interactions are implemented as possibilities of physical impact (ejection of an object); propagation of a number of chemical signals and removal (degradation) of the signal(s) over time. For each chemical signal, users can specify its primary spatial parameters, such as the significant action radius, signal propagation and signal degradation rates.

This accelerator is used in the mechanisms of coordinate selection in cell movement and axon growth. The *gradientCondition* is configurable by the user for a particular mechanism on a simulation setup.

**Algorithm 2:**
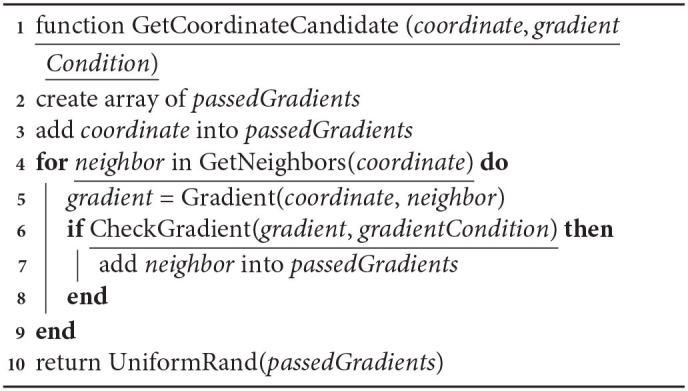
Algorithm of gradient checks. This function returns a satisfactory coordinate for gradient checks based on the user condition. Returns a randomly picked satisfactory coordinate.

### 2.3. Probabilistic Processes

Simulation is a powerful tool for approximated reproduction of complex processes. Simulation differs from emulation in that there is no need for detailed and precise repetition of all components of the process being reproduced: researchers can omit thorough descriptions of experimental conditions in favor of accelerated prototyping and refinement of hypotheses.

The BCNNM framework implements probabilistic execution of processes. The use of probability is mandatory to resolve interconnected and conflicting events, for instance in the case of two moving objects attempting to occupy the same coordinate. In addition, users are given the opportunity to configure a probabilistic execution of any signaling pathway in accordance with a specified distribution for a particular ligand in the spatial coordinate that is evaluated.

To obtain random variables in the simulation process, our framework uses a pseudo-random number generator (PRNG), which represents a function that produces a sequence of numbers. The sequences generated by the function will be unique and deterministic for various (user-defined) PRNG starting seeds, which ensures full reproducibility of the simulations.

### 2.4. Basic Implemented Mechanisms

The framework is based on a set of fundamental mechanisms such as biochemical signaling and diffusion, cell body interaction and stochasticity. Implementation of those mechanisms is premised on analytical formulas with adaptation to model assumptions such as discrete space and time. For instance, diffusion in the brain can be described with a modified diffusion equation (Sykova and Nicholson, 2008) and, taking into account the fact that in our framework only one object is allowed to occupy a voxel, we can simplify this equation to a convolution with discrete gaussian kernel. Signaling is based on the same 3D space filled with multiple factors and this information could be exploited in order to compute, for instance, a gradient that guides axon growth (Bhattacharjee and Folch, 2017). Not all processes can be simulated in a deterministic way either because of their stochastic nature or because they are too complex to be described in details. Cell division is a typical example of such an event, and it can be simulated using a probabilistic process (Ziebell et al., 2014; Bast et al., 2018) with multiple precision levels. In our framework probabilities can be specified by the user, taking either cell state or environment as parameters.

#### 2.4.1. Chemical Signal Secretion and Degradation

Extracellular chemical signals, which are secreted and degraded, are described as concentrations of signaling molecules (factors) in each spatial coordinate. Every cell type is described by two sets: *F*_*in*_—a set of factors, which the cells react to, and *F*_*out*_—a set of factors that the cells can release. For each accepted factor *f* ∈ *F*_*in*_ there is a non-empty set *SP*_*in*_ ⊂ *SP*, consisting of signaling pathways that are turned on in the case when the configured conditions are fulfilled. Such an approach allows for flexible settings in the final model.

Each factor *f*_*out*_ ∈ *F*_*out*_, emitted by a cell or a spatial mark, has its spreading limits called the emitted factor neighborhood *N*_*f*_ and is characterized by an emission radius *r*_*f*_, universal for a signal type. To simplify and speed up the calculations, the system does not take into consideration the signal outside the *N*_*f*_ neighborhood.

The following algorithm is used to calculate diffusion. The **signalPower** and **maxRadius** parameters are set by the user for each specific signal in the configuration for a particular simulation. Values are updated in the DES paradigm for each significant time point of the corresponding propagation, degradation or absorption calculation event.

**Algorithm 3:**
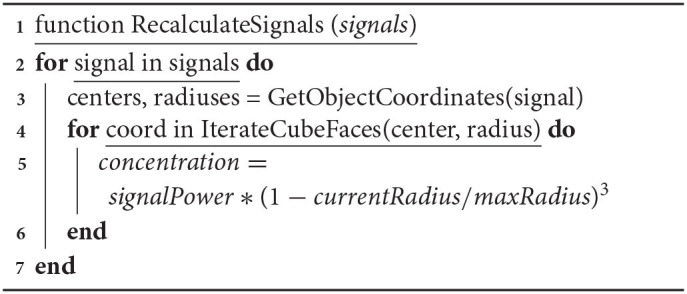
Algorithm of chemical signal secretion and degradation. Function is invoked for each iteration of spreading, gathering and degrading signal.

To validate the processes of diffusion implemented in our model we used the Gray-Scott equation with a single component as a reference, but the chemical reaction part was omitted because chemical interactions are not directly implemented in the current version of our framework. The parameters are: diffusion rate: 0.3, feed rate: 0.066, kill rate: 0.055, time 12.5 s. We compared the solution of this equation with a 2D slice of our model space (see Supplementary Figure 9).

#### 2.4.2. Signal Transduction

Signal transduction mechanism checks if specific conditions are fulfilled. These conditions can be configured individually for each SP in every compartment and cell type. Each condition can include both extracellular and intracellular sets of boundaries for enzymes, growth and differentiation factors, or other signaling molecules.

#### 2.4.3. Cell Locomotion

Movement is carried out in accordance with the algorithm A^*^ (Hart et al., 1968), where the minimum distance *l*_*min*_, by which an object can move, is the permissible unit offset *l*_1_, defined in the three-dimensional integer grid *L*. The neighborhood of all possible displacements is the *N*_*f*_, consisting of unoccupied coordinates of the *N*_*c*_. Upon selection of the transition coordinate, factor-dependent condition verification is carried out for *N*_*f*_. Each movement condition has a special rule concerning its direction: to follow either the chemical signal gradient or the anti gradient. The final displacement coordinate is chosen with equal probability from the positions of candidates remaining after all verifications.

#### 2.4.4. Proliferation

Cells are obviously intended to be of animal somatic origin, therefore by cell division we imply mitotic division. In our model, most of the details of this process (such as stages of mitosis) are omitted because their incorporation at this point would increase computational complexity without adding any useful functionality. Cell division in our framework is described as a separate mechanism; it can occur cyclically, in presence of growth factors or without them. When this mechanism is triggered, all the other signaling pathways in the cell are paused to allow for proper completion of the division process. Cells in this state can only be moved when displaced by other cells. After the division both daughter cells relaunch their initial SPs, appropriate for their respective cell types. Two types of division are implemented in the BCNNM framework: symmetric (both daughter cells are of the same cell type as the parental one) and asymmetric (one of the daughter cells is different: it has lower potency).

#### 2.4.5. Cell Differentiation

We implemented the differentiation process in accordance with the principles observed in living cells and a summary of neural cell lineage trees in mammals (Anderson, 2001; Carlson, 2014). In the BCNNM framework, cell differentiation is described as a separate mechanism. It occurs only if a certain condition is met. When this mechanism is triggered, all the other SPs in the cell are disrupted to allow for proper completion of the differentiation process. Cells in this state can only be moved when displaced by other cells. After the differentiation, cells initiate signaling pathways, appropriate for their new cell types.

#### 2.4.6. Cell Death

Apoptosis is described as a separate mechanism and occurs in the presence of certain factors as well as due to internal changes: concentration of the proteins responsible for cell death reach a critical concentration or changes occur in the parameters of particular cell types, i.e., incoming and outgoing synapse degradation. When this mechanism is triggered all the other SPs in the cell are terminated, removal of the cell from the system is irreversible. Cells in this state can only be moved if displaced by other cells. After the cell removal through apoptosis, nothing is released into the intercellular space, the surrounding cells do not experience any changes in their environment.

Necrosis is described similarly to the apoptosis mechanism with one exception: following the cell removal, parameters of the intercellular environment (concentrations of substances) change, which might affect the surrounding cells.

#### 2.4.7. Axon Guidance and Synaptogenesis

To date, a number of axon growth and guidance models have been developed (Goodhill, 2018; Druckenbrod et al., 2020), but some nuances of these processes are still uncovered (Stoeckli, 2018). One of the basic principles used in such models is the idea of following either a gradient or an anti-gradient of some chemical signal (Bicknell et al., 2018) that is used to control the axon movement.

In our framework the user is free to implement one of those algorithms or test a new one. We made the process of wiring fully configurable via using the framework pathways. In the specific models that we built in this study to demonstrate layer formation we used a simple gradient-based algorithm. *growthConditions* is a set of conditions over a subset of all user-defined signals. This set is specified by the user in the configuration for the axon growth mechanism of a particular simulation.

**Algorithm 4:**
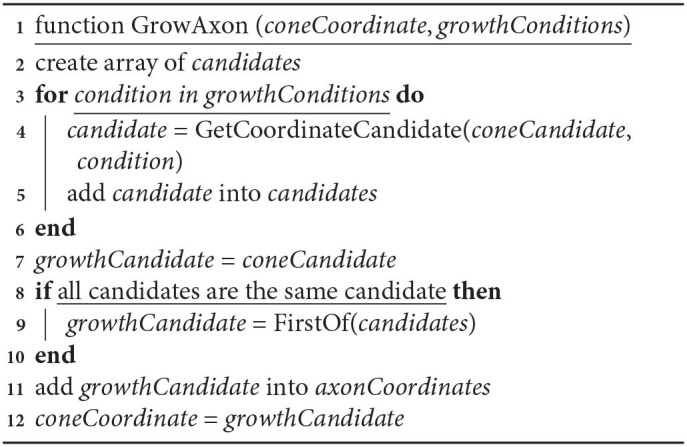
Algorithm of axon growth. For a “not growing” situation when there is no consensus on growth direction.

The process of synaptogenesis incorporates mechanisms of compartment formation, signal emission and the synapse formation stage itself. Axonal terminals, dendritic spines and synapses are formed one after another. Neurons that participate in the forming synapse exchange chemical messengers throughout the process to signal start or completion of their respective compartment creation events.

### 2.5. Experiment

This section presents a description of the experiments run on the basis of the outlined above framework. The experiments are designed and aimed to demonstrate the basic properties of the model, such as the ability to develop a layered interconnected structure composed of neurons and supported by various types of glial cells through the processes of division, migration and differentiation from a single initial pluripotent stem cell. The developmental progress is tightly regulated, but at the same time allows for certain flexibility in the input parameters, making the simulation process resilient and adjustable to the user needs.

The course of tissue development within the BCNNM framework can be formally divided into the following consequent stages:

- Stem and precursor cell proliferation;
- Precursor differentiation;
- Function-specific cell compartment formation;
- Axon growth and pathfinding, synapse formation.

Each of these steps is described in accordance with (and based on) the way similar processes occur in nature. Due to the fact that the details of biological processes referred to in this study are still being actively researched and, for most of them, science is still far from seeing the whole picture, the way they are implemented in our framework is simplified and conventionalized in order to make the model functional. It should be noted that regulatory factors mentioned in the diagrams and descriptions of the experimental part do not strictly correspond to any real-life biological molecules, these terms are used only within the BCNNM framework. The simulation process, in fact, is continuous and is not functionally divided into stages: we have broken it down in this article into four stages represented in the diagrams in order to make the perception easier.

#### 2.5.1. Stage 1: Precursor Cells Proliferation: Symmetric and Asymmetric Division

**Figure 2:**
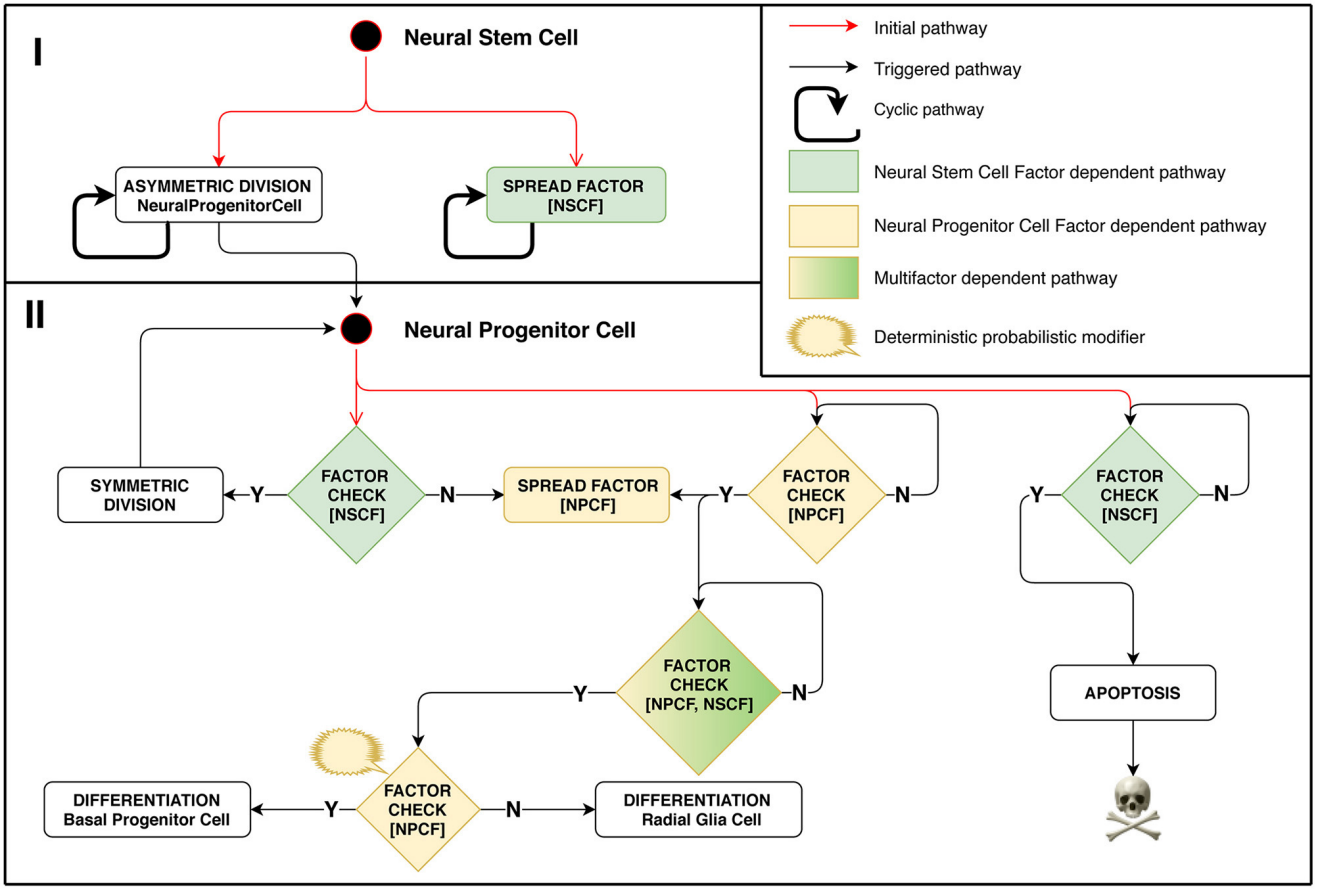
Stage 1: Role of chemical factors in stem cell behavior regulation. Different outcomes of the cell cycle shown depend on concentrations of factors distributed in space. Neural Stem Cell (NSC) is the starting cell of the simulation. NSC divides asymmetrically producing Neural Progenitor Cells (NPC) at regular time intervals. It emits Neural Stem Cell Factor (NSCF), which restricts the resulting structure size. NPCs divide symmetrically in presence of NSCF, yielding the exponential growth of the number of cells. The division is controlled by the NSCF signal value at the dividing NPC coordinate: if the value is small, it implies that the target structure size has been reached. In such a case, the NPC starts secreting the Neural Progenitor Cell Factor (NPCF). NPCs check for the presence of NPCF and a chain reaction of NPCF secretion is triggered in all NPCs of the cell agglomerate. The NPCF signal induces further probabilistic differentiation of NPCs into precursors of neurons and glial cells. Small NSCF value means that the NPC has been pushed out of the structure and is subject to apoptosis.

In this experimental setting, the division type for each stem cell is regulated by the biochemistry of its surrounding cells. This approach lets us mimic some aspects of tissue homeostasis that can be observed in biological systems. Model implementation of this stage is schematically presented in Figure 2. A positive feedback regulation loop is at work in case of Neural Progenitor Cells (NPC) located on the minimal meaningful concentration boundary of the differentiation-stimulating Neural Stem Cell Factor. They emit their own signal, Neural Progenitor Cell Factor (NPCF), which stimulates their surrounding cells to start secreting this factor as well, which triggers a chain reaction. NPC differentiation is initiated only when the NPCF concentration reaches a threshold value, which can happen if several closely located cells are engaged in factor secretion simultaneously.

#### 2.5.2. Stage 2: Differentiation Into Mature Cell Types

**Figure 3:**
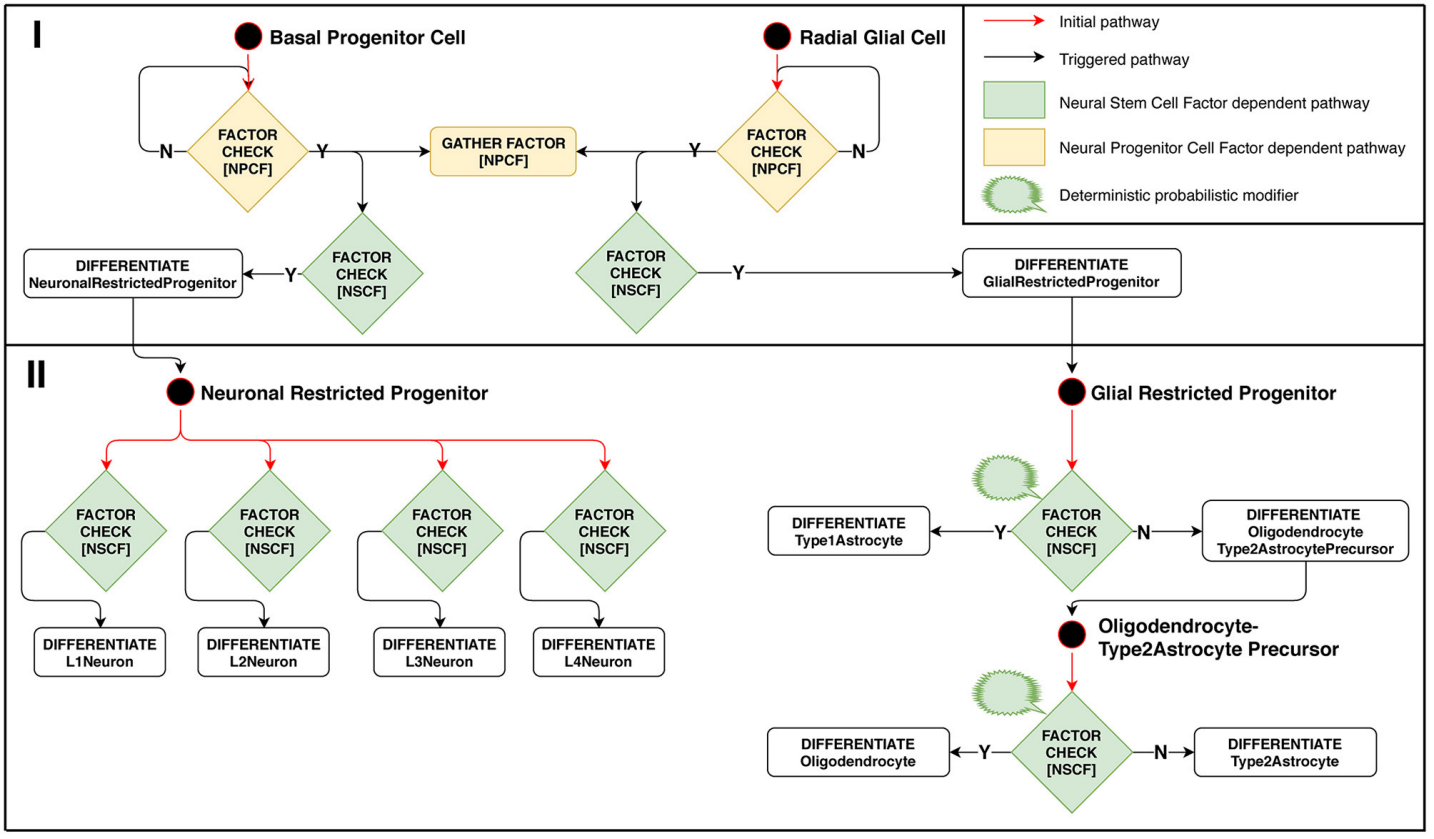
Stage 2: Factor-dependent differentiation of progenitors into neurons and glial cells. (Phase I): Basal progenitors and radial glial cells accumulate NPCF around themselves and differentiate in presence of NSCF. (Phase II): neuronal-restricted progenitors differentiate into different neuron types depending on the concentration of NSCF, forming a layered structure as a result. Glial-restricted progenitors differentiate probabilistically into different glia types in presence of Neural Stem Cell Factor.

Model implementation of this stage is schematically presented in Figure 3. The configuration for Neuron Restricted Progenitors differentiation into neurons is defined in such a way as to create overlapping layers: L1 → L2, L2 → L3, L3 → L4. Ranges of Neural Stem Cell Factor represent intersecting intervals in the differentiation rules.

#### 2.5.3. Stage 3: Axon and Dendritic Tree Formation

**Figure 4:**
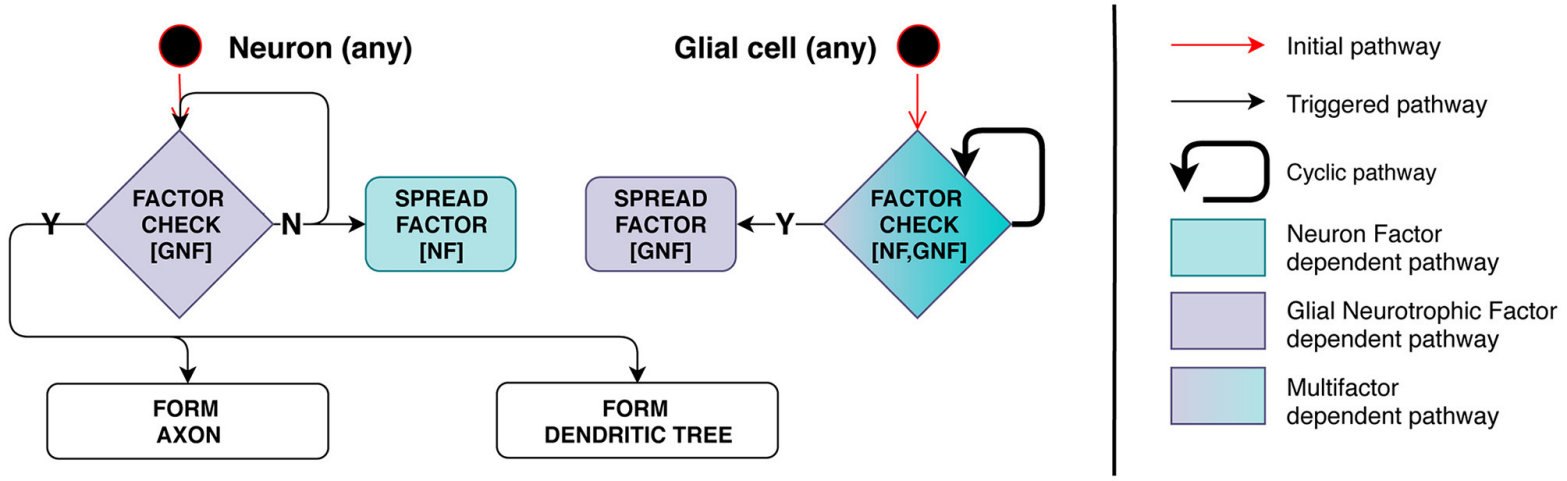
Stage 3: Interdependence of processes related to glia and neurons. On the **left**, neuron checks its surrounding space for presence of Glial Neurotrophic Factor (GNF). If GNF level is insufficient, the neuron emits Neuron Factor (NF) to inform glial cells of its presence. On the **right**, glial cell checks the surrounding space for presence of NF and measures GNF concentration. When the condition is met (enough NF and insufficient GNF is present), the cell starts secreting GNF. When the neuron detects GNF present in a sufficient quantity, axon and dendrite development is triggered and NF secretion is inhibited—negative feedback regulation is therefore employed.

Model implementation of this stage is schematically presented in Figure 4. Axon and dendrite formation occurs only in the presence of a sufficient amount of GNF, a factor secreted by glia. Therefore, this process depends on the number of mature glial cells in the immediate vicinity of the neuron.

#### 2.5.4. Stage 4: Axon Growth and Pathfinding, Synapse Formation

**Figure 5:**
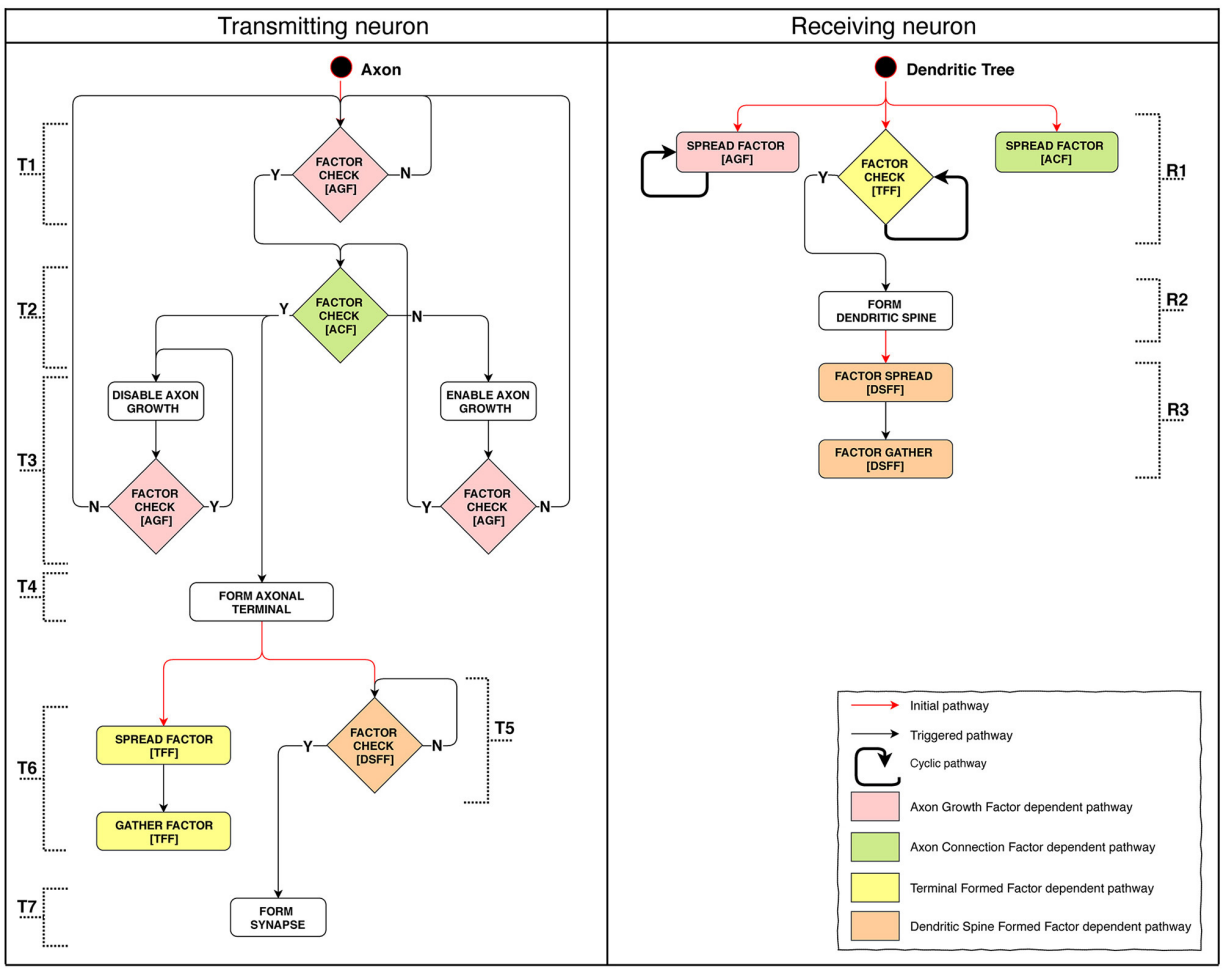
Stage 4: The process of axon pathfinding and creation of synapse. (T): (1) Axon's starting SP checks for Axon Growth Factor (AGF) and if it's there the Axon Connection Factor (ACF) presence test is launched, if not—the SP restarts itself. (2) If ACF is present: axon growth is arrested, axon terminal formation is launched. If ACF is absent: axon growth is launched. (3) Disabling growth makes axons switch direction to follow the reverse concentration gradient. It checks for AGF presence: if present, the SP restarts itself; if not, AGF availability check is launched (returns to the start). Enabling growth switches axon growth direction to follow the concentration gradient. It checks for AGF: if AGF is there, ACF presence check is launched; if absent, AGF presence check is launched (returns to the start). (4) Axon terminal formation. (5) DSFF presence (indicates the existence of a formed dendritic spine) check: if fulfilled, synapse formation starts; if not, the SP restarts itself. (6) TFF diffusion and subsequent absorption. (7) Synapse formation. (R): (1) Starting SPs of the tree: Axon Growth Factor (AGF) emission; Axon Connection Factor (ACF) emission; Check for presence of Terminal Formed Factor (TFF). (2) If TFF signal emitted by the axon terminal is sufficient, dendritic spine is formed. (3) First Dendritic Spine Complete Factor (DSCF) is emitted, then it is absorbed.

Model implementation of this stage is schematically presented in Figure 5. We were aiming to demonstrate the possibility to create connections between adjacent layers as well as across-layer. The connection arrangement is as follows: L1 → L3, L3 → L2, L2 → L4. The connection algorithm described for this stage does not directly restrict the possibility of creating repeated connections. After forming a terminal, the axon starts moving against the AGF factor concentration gradient. However, spatial patterns of concentration gradients can in some cases make the growth cone return to the neuron with which a connection has already been established. Because of this feature, we classify all connections into three categories: single, sequentially repeated and non-sequentially repeated outgoing connections (Supplementary Figure 2).

## 3. Results

For the computational experiment setup, 69 different configurations with descriptions of probabilistic processes were prepared. The configurations of experimental stages were tested out as separate sub-experiments to confirm the stability of mechanisms' operation. For the final comparison, in this section we present two configurations that differ in the differentiation rules for Neuron Restricted Progenitor Cells creating L3 and L4 neurons. For the ease of comparison, the two experiments run with these configurations (Configuration I and Configuration II) have been performed using the same PRNG seed. As a result of an experimental run, we obtain a fixed state of the model space and of a structure created within it, as well as exhaustive spatiotemporal information about the sequence of simulation events. This data allows us to analyze and compare the resulting networks. Structures with the characteristics listed in Table 1 have been built in the computational experiments with configurations I and II.

**Table 1:** Cell types and quantities at the end of the simulations.

**Cell type**	**Code**	**Configuration I**	**Configuration II**
**Multipotent Progenitors**	**MP**		
Neural Stem Cells	NSC	1	1
Neural Progenitor Cells	NPC	126	127
**MP total**		**127**	**128**
**Neuron Progenitors**	**NP**		
Basal Progenitor Cell	BPC	0	0
Neuron Restricted Progenitor Cell	NRPC	18 390	18 825
**NP total**		**18 390**	**18 825**
**Glial Progenitors**	**GP**		
Radial Glia Cell	RGC	0	0
Glial Restricted Progenitor Cell	GRPC	0	0
Oligodendrocyte-Type2Astrocyte Precursor Cell	OT2APC	0	0
**GP total**		**0**	**0**
**Neuron Cells**	**NC**		
L1Neuron (Afferent)	L1N	73 661	73 653
L2Neuron (Inter)	L2N	43 682	43 957
L3Neuron (Inter)	L3N	13 291	10 114
L4Neuron (Efferent)	L4N	381	2 823
**NC total**		**131 015**	**130 547**
**Glial Cells**	**GC**		
Type 1 Astrocyte	T1A	534 686	533 559
Oligodendrocyte	ODC	117 857	117 577
Type 2 Astrocyte	T2A	50 627	50 703
**GC total**		**703 170**	**701 839**
**All types**		**852 702**	**851 339**

**Figure 6:**
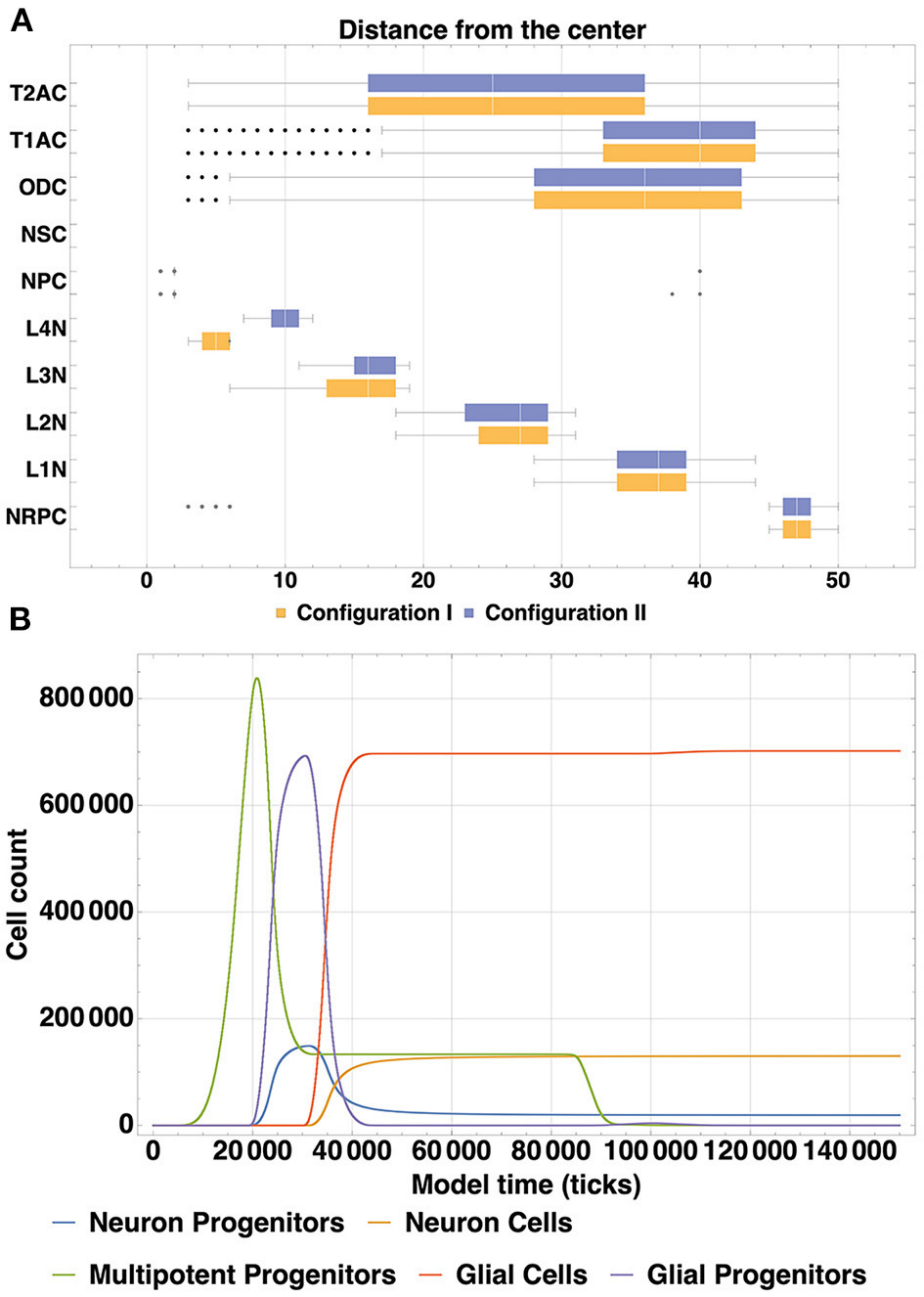
**(A)** Spatial distribution of all types of cells (Configuration I and Configuration II). A comparison of spatial distribution of different cell types acquired with two model configurations is shown: glial cells (T2AC, T1AC, and ODC), multipotent progenitors (NSC and NPC), neuron cells (L1-L4 neurons) and neuron restricted progenitor cells (NRPC) (Table 1). Differences in the structures are in the thickness of L3 (L3 in Configuration II is thicker than in Configuration I) and in the position of L4 (in Configuration I it is closer to the center than in Configuration II). In addition, there are some NRPCs around the symmetry center in Configuration II. Cells of other types are distributed similarly. **(B)** Dynamics of cell count within cell type clusters, based on the model Configuration II. The depicted dynamics of cell count is a consequence of cell division, differentiation and removal of cells in different groups: multipotent progenitors, neuron and glial progenitors, neuron and glial cells. The similarity of dynamics in analogous processes is shown, e.g., transition of progenitors to various mature cells types at 20.000–30.000 ticks, plateau phase starting at around 40.000 ticks after the active differentiation phase and mass apoptosis of multipotent progenitors at 69.000–90.000 ticks.

The spatial distribution of cells by type (Figure 6A) enables us to compare arrangements of the L1, L2, L3 and L4 cell types relative to each other in different configurations. The created structures differ from each other in positions and thickness of L3 and L4 layers: layer L3 obtained with Configuration I is thicker than L3 Configuration II, the boundaries between L3 and L4 Configuration I layers are distinct relative to each other; L4 Configuration II layer is located farther from the center than L4 Configuration I. Another dissimilarity between the Configurations II and I is the presence of neuronal restricted progenitors around the symmetry center in the former. Apart from that, cells are distributed in a very similar way.

The dynamics of cell count as a consequence of cell division, differentiation and removal of cells in different groups are depicted in Figure 6B. For analogous processes, substantial similarity can be observed in the overall shapes of their dynamics: cell number growth in different clusters demonstrates such resemblance, as well as cell differentiation progression (transition of progenitors to various mature cells types at 20.000–30.000 ticks). It can also be noted from the figure, that mass apoptosis of multipotent progenitors takes place at 69.000—90.000 ticks. It has been specified in the model configuration, that apoptosis occurs at the minimal concentration boundary of the NSCF growth factor chemical gradient, which has a spherical shape. Outer layers of such a sphere contain far more cells than the more central ones (due to simple geometry rules), which leads to the fact that numerous cells end up in these apoptosis-promoting conditions.

Neural cell cycle is highly variable between multiple cell types and environmental conditions and this leads to the diverse structures of neural tissue and a wide range of possible simulation results depending on model configuration. Stem cells (multipotent progenitors in terms of the BCNNM) and progenitor cells ratio during the first stage of the development in our simulation results (Figure 6B) exhibit dynamics similar to the experimental data on hippocampal neurogenesis reported in Ziebell et al. (2014): the ratio changes in time from 3.2 to 1.2 to 0.6 in our model and from 2.5 to 1.1 to 0.5 in the mentioned work. Temporal differences in cell lineage dynamics can be observed when compared to *in vivo* data: after the phase of symmetric proliferative divisions of multipotent progenitor cells in our model the neurogenic and gliogenic phases occur almost simultaneously (see Figure 6B), whereas in *in vivo* systems Radial Glial Progenitors (RGPs) first enter the neurogenic phase, after which the remaining (about 1/6) of RGPs enter the gliogenic phase (Supplementary Figure 4) (Gao et al., 2014; Beattie and Hippenmeyer, 2017). Such discrepancy is due to the way the cell lineage is designed in our framework: the population of multipotent progenitors corresponding to Radial Glial Progenitors (RGPs), after undergoing proliferative symmetric divisions, specialize, and become more restricted in their potency—destined to become either neurons or glia. In such a way, early specialization of the progenitors occurs instead of the temporal shift, and neuro- and gliogenesis proceed simultaneously.

To analyze the formation of layers L1-L4, a plot depicting differentiation of neuronal restricted progenitors into corresponding neuron types was created (Supplementary Figure 3). The figure demonstrates the fact that differentiation occurred sequentially: first, the L1 cells differentiated, then the L2, L3, and L4 cells. In addition, the graphs for the L1-L3 layers show that upon reaching a certain quantitative indicator, the number of cells of each of these types becomes relatively constant. This observation can be explained by the fact that in the zone where these cells are located, no precursor cells capable of differentiating are available any longer.

The character of the L4 layer curve does not lead us to a similar conclusion. The reason for such dissimilarity in behavior is rooted in the layer's location: it is close to the center. The cross section in Figure 8 shows a cluster of neuronal restricted progenitors in the center of the structure, and their potential for differentiation does not run out until the end of the simulation.

**Figure 7:**
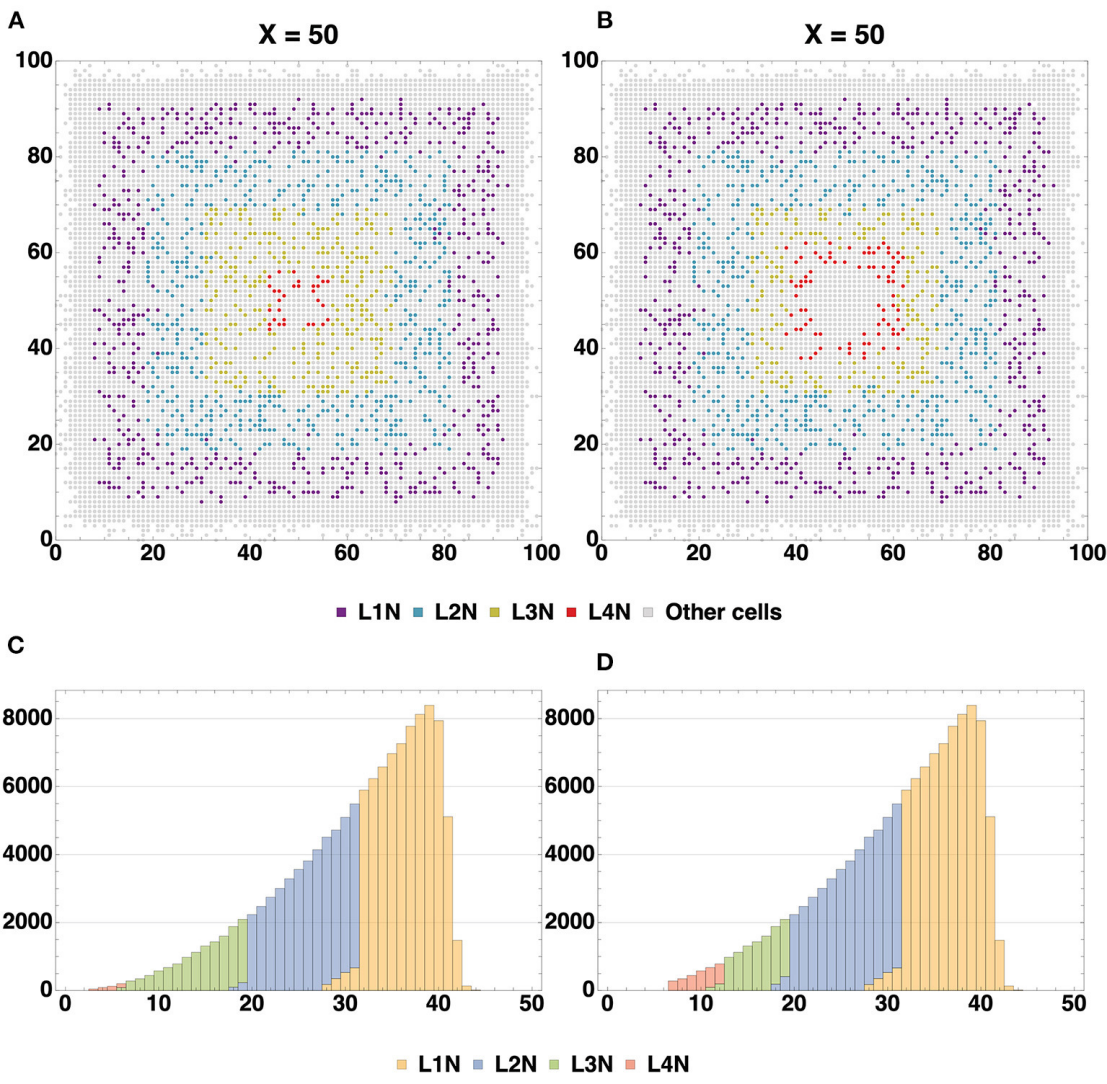
Cross-section of the resulting structure with all neuron types highlighted, based on Configurations I **(A)** and II **(B)**. Distribution histograms for L1-L4Neuron cells in Configurations I **(C)** and II **(D)**. The total count of all neurons (L1-L4) varies insignificantly between the Configurations I and II (about 500 per 130 k—0.358%), while the number of cells in the L3 and L4 layers does vary to a greater extent: modeling with Configuration II resulted in both L4 overgrowth and L3 thinning compared to Configuration I (0.011 and 0.626% for L1 and L2; 31.4 and 86.5% for L3 and L4). Additionally, it can be seen that at the plateau stage the model space is not completely filled with cells.

The formed neuron layers as well as the differences between Configurations I and II can be clearly seen in cross sections (Figures 7A,B). The histograms (Figures 7C,D) show the spatial distribution of neuron types L1-L4. The primary distinction is that the Configuration II L4 layer is located farther from the center, and the increase in the number of L4 cells is “at the expense” of the L3 layer: in the Configuration II L3 contains fewer cells than that in the Configuration I. The total number of all neurons (L1-L4) varies insignificantly between the Configurations I and II (about 500 per 130 k—0.358%), while the number of cells in the L3 and L4 layers does vary at a greater range (0.011 and 0.626% for L1 and L2; 31.4 and 86.5% for L3 and L4). These changes are due to altered marginal concentrations of Neural Stem Cell Factor in the rules for differentiation of Neuron Progenitor Cells into L3Neuron and L4Neuron in different configurations.

**Figure 8:**
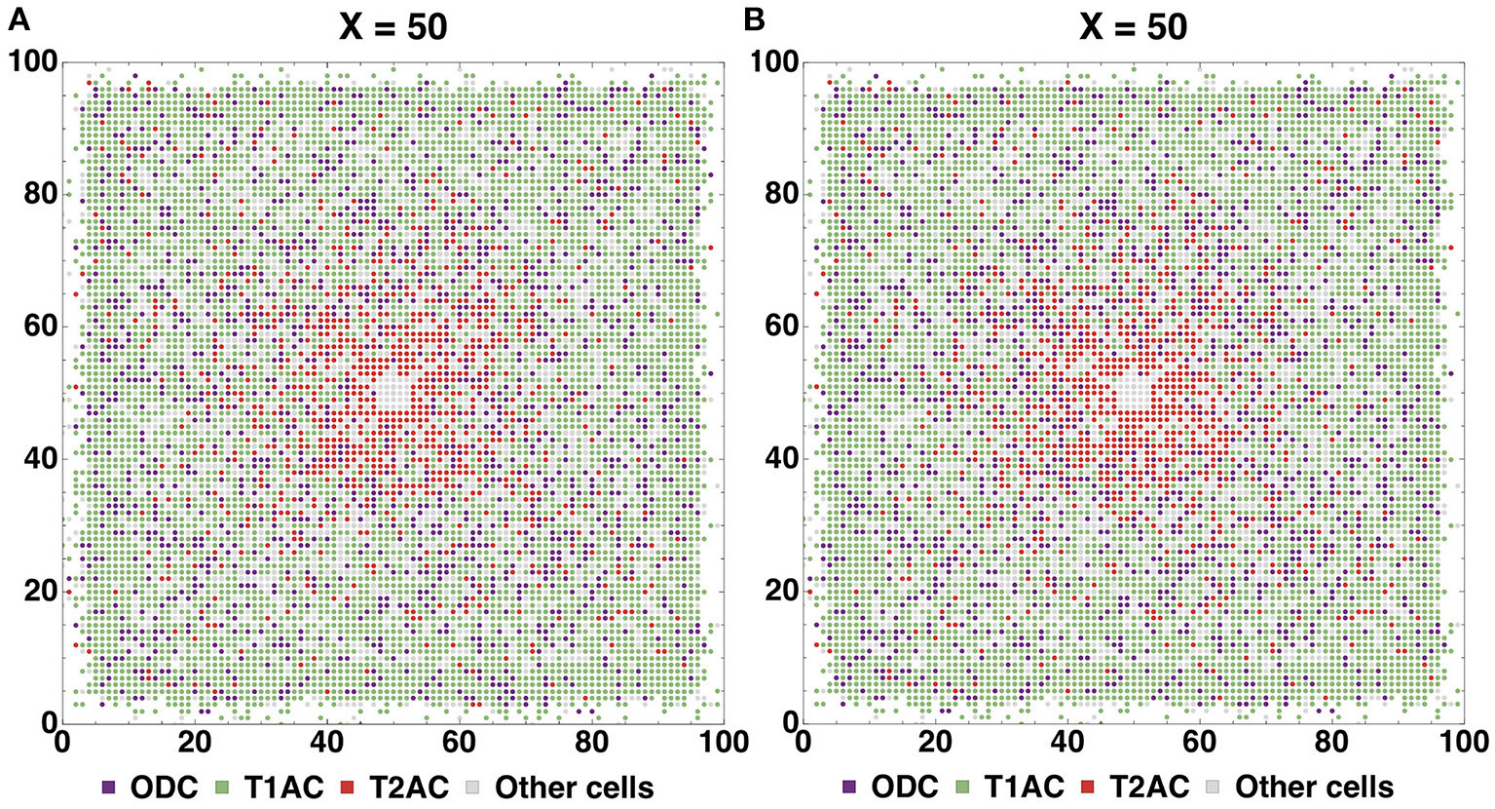
Cross sections for glial cells based on Configurations I **(A)** and II **(B)**. The distribution of glial cells is such that the density of T1A cells is higher in the center, while T2A cells are more densely spaced closer to the periphery of the simulated organoid. OD cells are spaced evenly.

Cross sections for glial cells shown on (Figures 8A,B). The distribution of glial cells is such that the density of T1A cells is higher in the center, while T2A cells are more densely spaced closer to the periphery of the simulated organoid. OD cells are spaced evenly. Since we did not change rules for glial cell density distribution between these configurations, the cross sections are nearly identical.

These results can be validated by experimental data both from organoid culture assays and *in vivo* mammalian brain studies. The layered structure which was produced in our experiment is intended to be a generalized representation of organoid architecture (rather than any particular brain structure like neocortex), with the potential to be tweaked to achieve higher degree of similarity to any particular region of interest. Despite some discrepancies in the number and shapes of layers, spatial distribution of cells and relative positions of layers have a comparable arrangement to the parameters observed in *in vitro* three-dimensional neural spheroid cultures (Dingle et al., 2015). In order to further validate the layer structure created in our model, we compared the distribution of normalized distances from the center for different Neuron cell types with analogous parameters (distance from the intermediate zone between the ventricular zone and cortical plate) from Caffrey et al. (2014). Even though our model has fewer layers (4 vs. 6 in Caffrey et al., 2014), L1, L2, L3, and L4 have similar spatial alignment and distribution.

**Figure 9:**
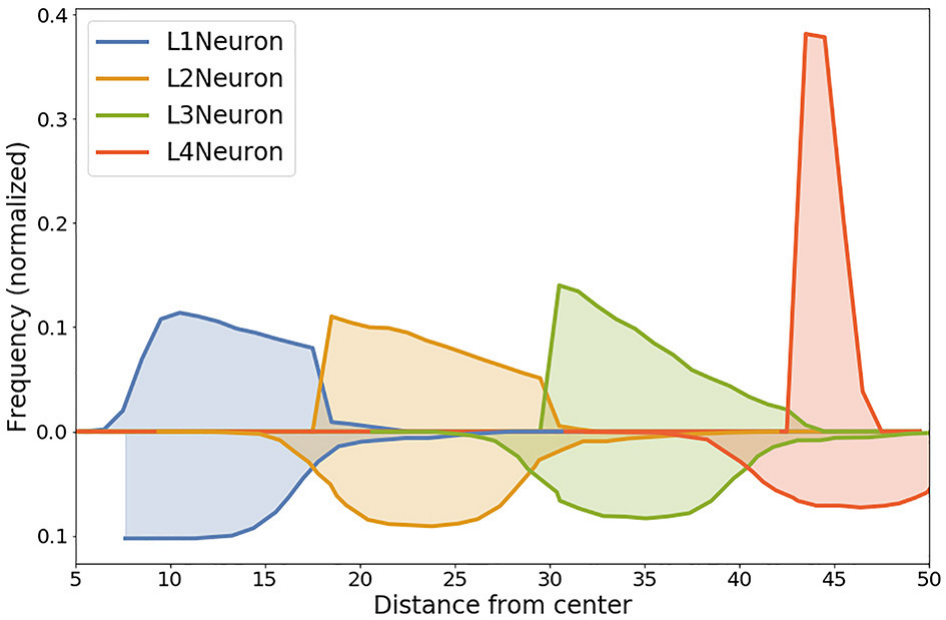
Comparison of the distribution of normalized distances from the center for different Neuron cell types in our model with experimentally measured distances from the intermediate zone between the ventricular zone and cortical plate from Caffrey et al. (2014). Data from the simulation is shown with lines above the x-axis, experimental data is shown lines below the x-axis. High level of correlation can be observed for layers L1, L2 and L3. Entropy values of the corresponding distributions: L1—0.11, L2—0.26, L3—0.38, L4—1.77.

Visual representation of the comparison is presented in Figure 9: lines below X axis in the plot represent distribution of cell positions in the (Caffrey et al., 2014) model, lines above X axis—distribution of cells positions in our simulation. Entropy values of the corresponding distributions for cells in L1, L2, L3, L4 layers are 0.11, 0.26, 0.38, and 1.77, respectively.

The cross section (Supplementary Figure 5) shows the locations of glial cells. Type2 astrocytes are distributed with greater density toward the symmetry center, where they are more common than other glia types. Type1 astrocytes demonstrate the highest abundance of all glial cell types and they are found in large numbers close to the surface of the tissue and are less frequent in the central zone. Oligodendrocytes are scattered evenly throughout the whole structure, except for the centermost coordinates, where only neural progenitor cells are present. In accordance with the rules guiding the experimental Stage 3, glia affects the formation of axons and dendrites. Because of this, the quantitative ratio of glia and neurons has to fit into a certain range in order to facilitate further successful creation of connections. Table 2 lists quantitative ratios of all glial cell types relative to all neuronal cells within a respective layer which they occupy.

**Table 2:** Ratio of glial cells (all types) to neurons.

**Layer**	**Configuration I**	**Configuration II**
L1	6.06	6.07
L2	3.62	3.6
L3	3.33	3.77
L4	4.22	3.65

**Figure 10:**
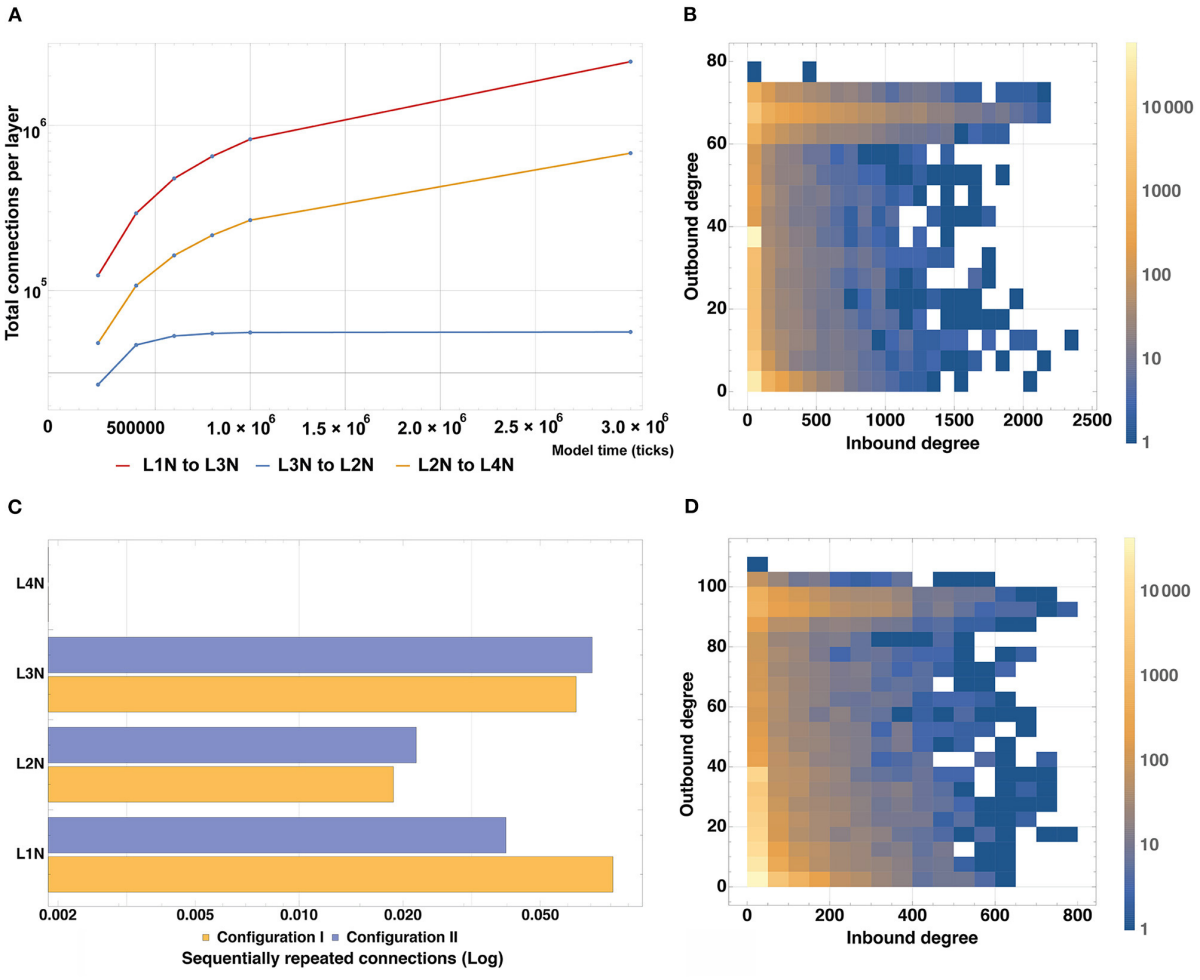
Model structure connectivity characteristics. **(A)** A number of connections between layers as a function of the simulation time based on Configuration II. The number of connections in the pairs L1-L3 and L3-L2 increases steadily with time and does not reach a plateau within the modeling timeframe, whereas the L2-L4 pair reaches its plateau quickly (1.0 × 10^6^ model ticks). **(B)** Quantitative distribution of connections for all types of neurons based on Configuration I. Most neurons have up to 40 outgoing and up to 100 incoming connections whereas the maximum outgoing rate is 80 and the maximum incoming rate is 2,400. **(C)** Number of sequentially repeated connections for Configuration I and Configuration II. For L1 and L3, the level of sequentially repeated connections stays above 0.042, while for L2 it stays under 0.028 for both Configuration I and II. **(D)** Quantitative distribution of connections for all types of neurons based on Configuration II. Most neurons have up to 20 outgoing and up to 50 incoming connections, the outgoing maximum is 110, the incoming maximum is 800.

To analyze the connectivity of the modeled structure, we plotted changes in the number of incoming and outgoing connections for neurons of each type in the Configuration II experiment with time (Figure 10A). According to the connectivity scheme described in the Stage 4 of the experiment, Configurations I and II predicate the following “outgoing-incoming” pairs: L1-L3, L3-L2, L2-L4. As can be seen in the graphs A and B, the number of incoming connections matches the number of outgoing ones when considering the corresponding type pairs. It is also possible to observe the varied dynamics of establishing new connections as the simulation period increases: the number of connections in the pairs L1-L3 and L3-L2 increases steadily with time and does not reach a plateau within the longest modeling timeframe, whereas the L2-L4 pair reaches its plateau quickly (1.0 × 10^6^ model ticks).

To assess and compare the total number of incoming and outgoing connections and their affiliation, heatmap graphs for Configurations I and II were constructed (Figures 10C,D). They show the quantitative distribution of connections, as well as their maxima for all types of neurons. For the Configuration I, it can be seen that most neurons have up to 40 outgoing and up to 100 incoming connections, whereas the maximum outgoing is 80 and the maximum incoming is 2400. For the Configuration II, most neurons have up to 20 outgoing and up to 50 incoming connections, the outgoing maximum is 110, the incoming is 800. A graph showing the number of sequentially repeated connections is presented in Figure 10B. When comparing the two configurations, it can be seen that L2 and L3 Configuration II neurons establish more sequentially repeated connections than those in Configuration I, whereas L1 neurons establish fewer.

The spatial distribution of neurons that possess at least one outgoing or incoming connection is presented in Supplementary Figure 6. When comparing the obtained results, it can be noted that the most distant L1 neurons in the Configuration I experiment do not create connections, which is directly caused by the concentration parameters of ligands emitted by the L3 neurons. We also observe a dissimilarity between the distributions of L2Neuron with established outgoing connections and L2Neuron in general. These differences can be directly linked to the concentration parameters of ligands emitted by the types of neurons they target in axon guidance.

We run our model for both configurations 10 times with different seeds for random number generator and incorporated the aggregated statistics. Table 3 lists connectivity characteristics. The rows “axon/dendrite connection rate” contain mean and standard deviation for proportions of neurons in a layer that have established one or more outgoing/incoming connections relative to the number of neurons (in the same layer) that have successfully formed the corresponding compartment. In the “Interneuron rate” row, mean and standard deviation for proportions of neurons that have both incoming and outgoing connections out of the number of neurons with formed compartments are given. As can be seen in the table, Configuration I demonstrates overall lower connectivity if compared to Configuration II: especially pronounced is the difference in the L2-L4 pair and in L2Neuron, for which the Interneuron rate is off by 22%.

**Table 3:** Connectivity of neural structure. Configuration I and II.

	**L1 Neuron**	**L2 Neuron**	**L3 Neuron**	**L4 Neuron**
**Configuration 1**				
Neuron count	73869.7 ± 248.95	43880.2 ± 211.37	13387 ± 126.61	406.1 ± 25.03
Axon connection rate	0.99 ± 0.001	0.511 ± 0.024	0.996 ± 0.001	n/a
Interneuron connection rate	n/a	0.412 ± 0.02	0.991 ± 0.001	n/a
Dendrite connection rate	n/a	0.809 ± 0.009	0.995 ± 0.001	0.986 ± 0.009
**Configuration 2**				
Neuron count	73869.7 ± 256.66	44132.4 ± 234.4	10142.1 ± 91.65	2908.2 ± 42.26
Axon connection rate	0.915 ± 0.003	0.742 ± 0.007	0.988 ± 0.003	n/a
Interneuron connection rate	n/a	0.607 ± 0.014	0.983 ± 0.003	n/a
Dendrite connection rate	n/a	0.826 ± 0.016	0.995 ± 0.001	0.999 ± 0.001

Differences in the connectivity of the structures obtained with the two configurations, which are presented in Figure 10B and Supplementary Figure 6 and in Table 3, are caused by the spatial arrangement of the neuron types (shown in the analysis of cell distribution by type), as well as by the unequal significant propagation radii of the ligands responsible for axon guidance (L2NeuronAGF radius in the Configuration I is 1.5 times larger than in the Configuration II; L3NeuronAGF radius in the Configuration I is smaller only by 1 coordinate than in the Configuration II; L4NeuronAGF radius in the Configuration I is 1.4 times larger than in the Configuration II). These radii were configured individually for each ligand and every neural cell type, therefore we observe varying results not only in case of the L2-L4 pair, but for other neuron types as well.

**Figure 11:**
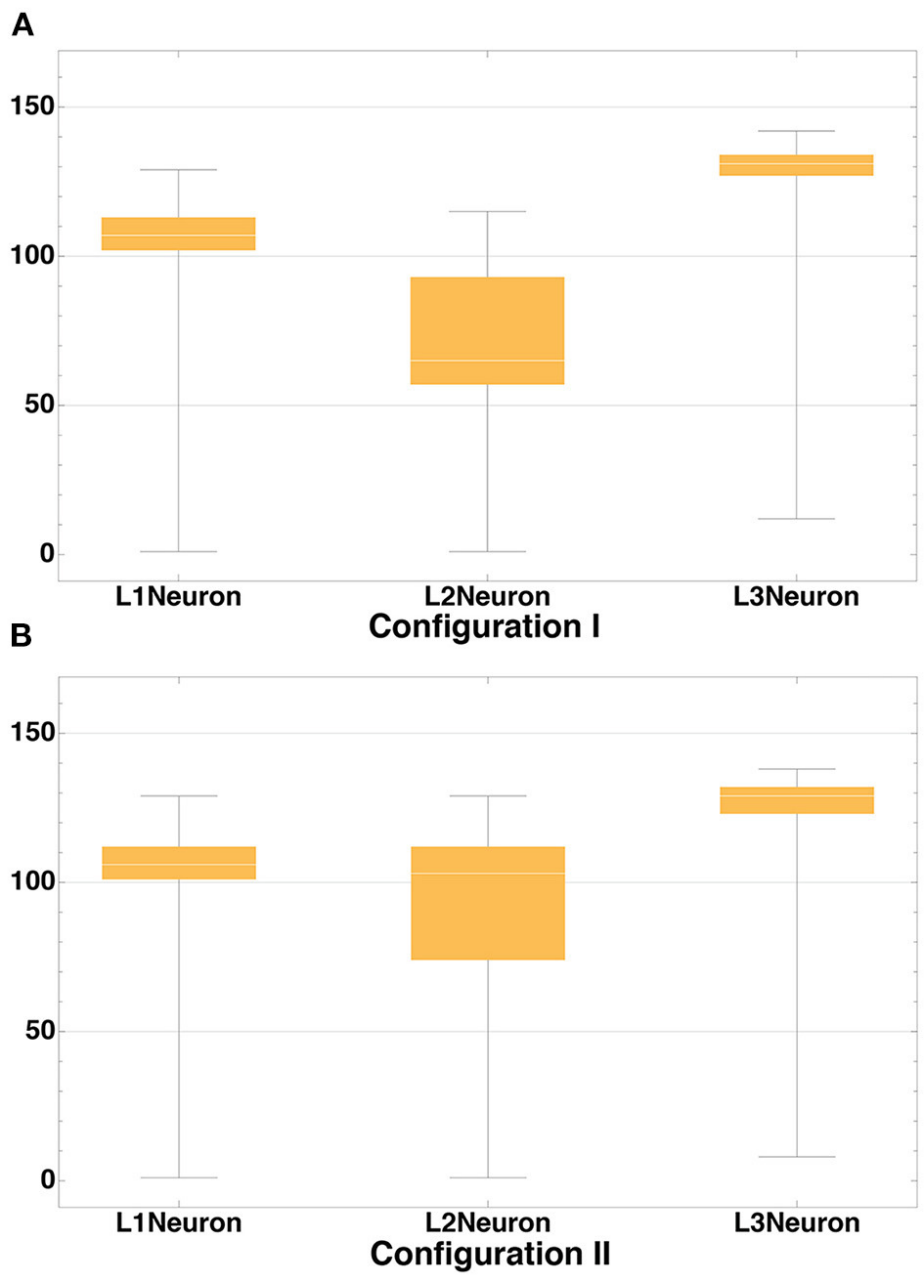
Axon length distribution. Axon length distribution for layers L1-L3 for Configuration I **(A)** and Configuration II **(B)**. In Configuration I the total length of axons of neurons located in the layers L1 and L3 is significantly greater than that in L2. In Configuration II this value is approximately the same.

**Figure 12:**
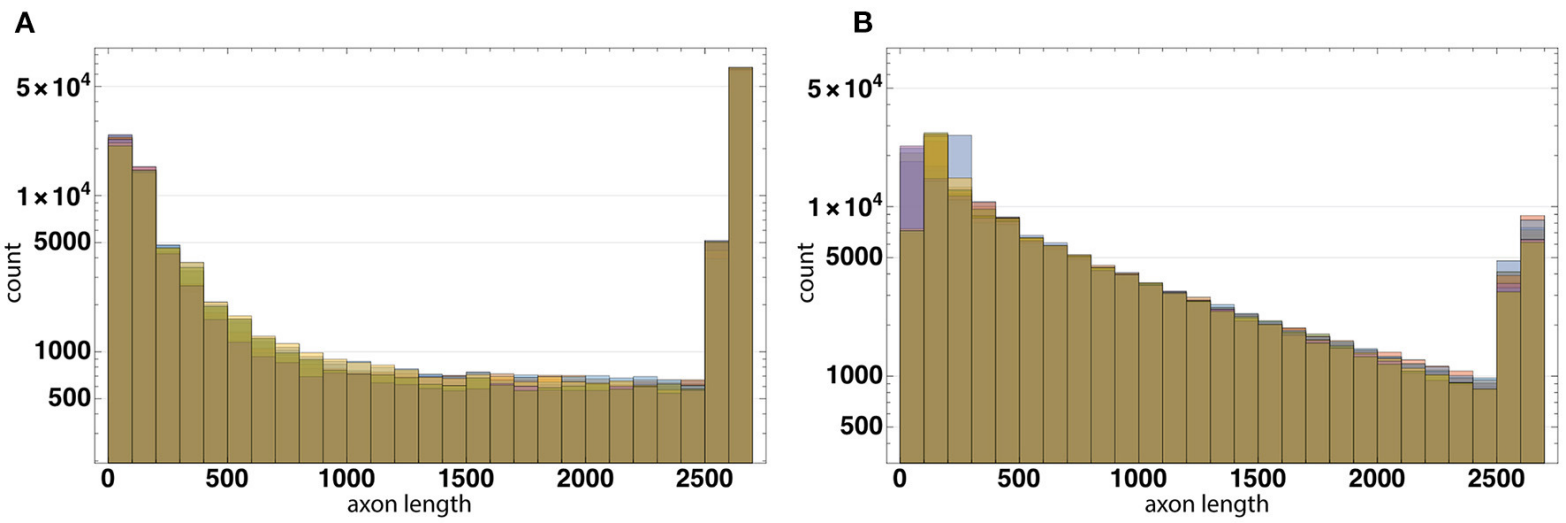
The distributions of the ratios of axon length to the number of axons for 10 runs of each configuration based on Configurations I **(A)** and II **(B)**. The distributions are different between the configurations, while between the runs of each one with different random seeds they remain highly similar. These results show that the model performance depends more on the configuration rather than on the random number generator.

The distribution of axon lengths for each layer is shown in the (Figures 11A,B). The distributions of the ratios of axon length to the number of axons for 10 runs of each configuration are shown in the (Figures 12A,B). The distributions are different between the configurations, while between the runs of each one with different random seeds they remain highly similar. These results show that the model performance depends more on the configuration rather than on the random number generator.

Neuronal connectivity in the presented computational models displays patterns similar to those observed in real structures. We have also compared our connectivity parameters with those of modeled neuronal structures from other *in silico* studies. For instance, a model designed specifically to simulate network growth (van Ooyen et al., 2014) yields consistent resulting connectivity parameters to the ones observed in our structures: when we compare parameters of L1/L2/L3/L4 Neurons from our model with those of apical cells from van Ooyen et al. (2014), outbound degree mean is 24.2 (±SD 19.9) and 21.7 (±SD 20.2) connections per cell, respectively, inbound degree mean is 24.2 (±SD 113.7) vs. 21.7 (±SD 21.6) connections per cell, respectively. This is consistent with the experimental data from Gerhard et al. (2011) (entropy for distributions of outbound degree is 0.57, mean of outbound degree 4.9), but our model contains cells with high density of connections (>10), which explains the higher mean value.

## 4. Discussion

Here we introduce a framework for dynamic simulations of biological mechanisms underlying the formation of organized neural multicellular structures (cerebral organoids) with hundreds of thousands of cells and millions of synapses. We have demonstrated the potential for using this framework to develop various spatial arrangements of cells, working at different approximations. These approximations, even though quite simple, provide a solution highly similar to the analytical one. The seemingly noticeable difference on the diffusion gradient shape is attributed to the fact that in the BCNNM we use non-euclidean Chebyshev space where a square diagonal is equal to its side, which makes circles look more like squares (see Supplementary Figure 9).

We have acquired a composite multicellular structure through a series of computational experiments (Figures 2–5), and recapitulated many basic principles of *in vitro* development of organoids and containing a complex cell lineage (Carlson, 2014), which is based on biological neural cell lineage data and adapted for *in silico* experiments. The resulting agglomerate contains up to one million cells (Table 1), which, if differentiated correctly, self-organize into an interconnected layered structure (Figures 7C,D and Supplementary Figure 6), containing axons and millions of synapses within and between different layers (Figures 10B–D and Table 3). The structure and its arrangement is similar to other *in silico* modeling experiments (Caffrey et al., 2014), as well as *in vitro* assays (Dingle et al., 2015) in terms of the overall layer ordering and characteristics like the distribution of normalized distances from the center for different cell types (for details see the section 3). In addition, we have shown the possibility to achieve any desired total cell count and ratios of cell types (Table 2) through adjustment of the differentiation rules, including fine-tuning of the numerical values of factor concentrations, as well as characteristics of chemical signal level probability distributions.

The mechanisms of apoptosis and restriction of cell division in the presence or absence of specific regulatory factors were successfully employed and we show how the total cell number is limited by these factors (see figure describing Stage 1 of the Experiment (Figure 2). Due to the imposed constraints, the model space does not end up completely filled with cells (Figure 7 and Supplementary Figure 5). This was performed in such a way that cells located at the boundary of significant concentration of the division-promoting factor (NSCF) terminate their division process, while those positioned beyond this boundary initiate apoptosis. These features are key to many stages of tissue formation, since various combinations of chemical gradients determine the behavior of cell groupings based on paracrine, autocrine, and other types of signaling. Furthermore, the use of varying signal propagation radii, significant concentration thresholds, and combinations of conditions has allowed us to establish the general direction of the differentiation process (e.g., inside-out or outside-in) within the cellular agglomerate in accordance with the needs of the experimenter (Figures 6A, 7A,B). Connectivity parameters are fundamental descriptors of any neural network and the number of outbound and inbound connections is one of the basic features characterizing a functioning neural cell. In our computational experiment, we observed connectivity characteristics (inbound degree, outbound degree, density of connections) similar to those documented in some real structures, for instance, cortical networks extracted from multi-electrode recordings of the visual system of a monkey (Gerhard et al., 2011), as well as to simulation results from other computational research (van Ooyen et al., 2014) (for details see the section 3). All of the mentioned features enable the user to create *in silico* complex multicellular neural organoid-like formations with a range of variable parameters such as the size, cell type ratios, presence/absence of layers, number of layers, connectivity, while closely monitoring their development.

Configuration flexibility is one of the advantages of our framework. A complex developmental pattern of the simulated object is achieved through combinations of simple mechanisms and rules for their interaction (Supplementary Figures 7, 8). The user interacts with the model configuration only, without the need to edit the source code. The configuration of the platform operates with standard biological concepts, such as cells, compartments, and signaling pathways among others. Individual simple mechanisms of the model are configured into more complex signaling pathways. Conditions that trigger certain signaling pathways are based on biochemical interactions, which allows us to simulate a dynamic system with complex regulatory feedback loops. In effect, the configuration is a collection of biological data on the basis of which hypotheses are formulated.

Formalism as an approach empowers the user to configure a large variety of experiments, but not simply in any piece of *ad-hoc* code. This software does not allow users to create improvised mechanism implementations by themselves, instead there is certain freedom to configure built-in mechanisms, such as cell signal propagation, differentiation, migration and so on. Using these basic mechanisms and configurations of varying complexity very different experiments can be built. An example of the simplest kind of such a biological experiment would be observing the growth rate in a culture of NSCs. After the onset of symmetric divisions in the modeled culture, the shape of the growth curve mimics the logarithmic growth phase observed in live cell cultures (Zheng et al., 2006; Li et al., 2017). Further restriction of the division and culture growth process in the model is achieved by means of mechanical factors (limited space) and chemical signals that incur differentiation, thereby creating the characteristic S-shape of the curve. Another simple example would be a cell culture response to a morphogen gradient. As is was shown, for instance, by O'Grady et al. (2019) in a tightly controlled *in vitro* assay, a differentiation cline is formed by cells along the gradient of a morphogen and it can be artificially tweaked by changing where the source of the morphogen is or by introducing additional sources. Exactly the same can be achieved in our framework and the differentiation cline patterns that we observe are remarkably similar (an example of such a cline in the presented experiment can be seen in Figures 7A,B).

The BCNNM framework occupies a niche between highly detailed dynamic models of processes in individual cells or in small populations of cells (Caffrey et al., 2014; Giacomantonio and Goodhill, 2014; Padmanabhan and Goodhill, 2018) and statistical models of very large cell populations, which, thanks to the simplified descriptions of low-level processes, make it possible to represent the collective behavior of tens of millions of cells (Gohlke et al., 2007; Borisyuk et al., 2011; Ziebell et al., 2014; Beccari et al., 2017; Razetti et al., 2018). The approach employed in the BCNNM enables us to build models successfully at the level of both cells and tissues or organoids, which allows for quick scaling of the intended research. As a result, even when dealing with millions of objects (cells, axons, dendrites, synapses), one can trace all individual state changes for each and every object in the simulation (Supplementary Material: Data S2). These state changes inside the model correspond to processes of different levels occurring in real living systems (e.g., cell division, cell death, emission of soluble signals, etc.). Thus, the BCNNM framework can be used for thorough *in silico* replication of *in vitro* experiments aimed to obtain complete sets of measurements from all key components, as well as for preliminary computational testing of novel hypotheses.

Another asset of this model is that the user does not need to have specific programming knowledge or skills to operate within the framework. The user is involved at a high level and forms their own hypotheses, wrapping them into the starting configuration of the model. Parameters of specific cells and signaling pathways are available for editing in the configuration file, which simplifies the process and allows the user to figure out quickly, how changing any particular parameters would affect the final result. With modern multi-core computers, it is possible to run several instances of configurations simultaneously, which allows for faster testing of hypotheses, for example, the computation of the presented in the experimental section takes about 4 h (for details see Table 3). The user does not need to rebuild the model from the source code when changing the parameters, which greatly improves the user experience. The framework demonstrates high performance (Supplementary Table 1), and the load depends only on the selected level of detail and scale of the model.

For the purpose of putting further constraints on the model with experimental data, tho general approaches might be taken. The first option is to use already published *in vitro* or *in vivo* results and build model configurations in such a way as to replicate the setup as closely as possible, at least in those aspects that are essential for observing an outcome on the parameters of interest. We are currently working on replicating some of the quantitative results obtained in a study of the deterministic progenitor behavior and unitary production of neurons in the neocortex by Gao et al. (2014): we aim to determine whether the RGP clone size distribution and probability of exiting the cell cycle in a model setting would align with what was found in this research to produce neuron counts and distribution expected for the given type of cortical structure. A very different verification approach would be designing *in silico* and *in vitro* assays in parallel. For instance, we could grow cerebral organoids *in silico* using predetermined sets of model conditions and acquire cell count (and possible connectivity) statistics, while simultaneously producing *in vitro* organoids in comparable real life conditions. qPCR and immunocytochemistry methods can then be used to asses quantitative parameters of these cellular structures, which would be compared with *in silico* data for model verification. At this point we do not have the ability to carry out wet lab experimentation, but we hope to achieve that through a collaboration in the nearest future.

It is important to keep in mind that organoids themselves represent simplified models of complex *in vivo* structures, which means that even the highest-fidelity simulations of their development will inherently be subject to both the limitations of the modeling approach and those of the organoid culturing method. Another potential shortcoming of our current modeling strategy is that neither cells nor their processes (axons) are attributed physical dimensions: this characteristic will be incorporated as soon as we notice that its absence renders our results biologically inaccurate.

Osmotic processes and cell volume changes are biophysical phenomena that are tightly linked with both tissue development and functioning. In the current version of the framework the simplification level did not allow us to take these aspects into account, but we are currently working on a more efficient build, which is capable of handling osmosis and cell volumes alongside with neural spiking. Neuron activation is implemented in the current version, although it is still in development and it is not presented in this study. Electrical signaling is the cornerstone property of neural tissue and the basis of information transfer in the brain. Spiking neural network models can be used as a bridge to connect the advances in computer science with biology and may serve as outcome predictors for *in vitro* experimental protocols (Khalil et al., 2017, 2018). We are going to discuss the applications of this model functionality in a separate publication.

The upcoming developments of the BCNNM framework can be divided into two major categories: (1) design and setup of additional *in silico* experiments, refinement and verification of the model accuracy using *in vitro* assay data, (2) further functional upgrades of the framework aiming to allow for more complex and detailed experiments. With the current version of the BCNNM framework, it is possible to simulate processes involved in mechanical damage of tissues that have a necrotizing effect, and the regenerative processes that follow it. Experiments can be set up to study post-traumatic recovery of nerve tissues from the structural (allows for comparison of tissue constitution before injury and after recovery) and functional (comparison of signal conduction functioning, repeatability of neuron excitation patterns) perspectives. Further work in this direction will potentially allow us to gain insight into underlying mechanisms of pathogenesis in various neurodevelopmental disorders, especially once we incorporate the finer probabilistic regulation mechanisms based on molar concentrations and multi-component signaling pathways. We are planning to refine the model description of programmed cell death as a mechanism limiting the resulting cell count as well as participating in the establishment of functional neuronal circuits. In order to set up experiments on axon growth regulation in our framework, it is necessary to integrate the ability for a neuron to emit signals communicating the number of established incoming connections. The probability of synapse formation with such neurons should be adjusted via autocrine signaling. In this way, the balance in processes of growth and synapse formation would be maintained at the tissue level, since the neurons would be making decisions based on the total number of neuronal connections in any given neighborhood. The following neurotransmission-related mechanisms are implemented in our framework; neurotransmitter synthesis, release of a particular amount of neurotransmitter into the synaptic cleft, stimulation of the postsynaptic neuron, reuptake and leakage of neurotransmitter. Functioning of the mentioned above processes is not described in this article, because simultaneous demonstration of all the mechanisms of the framework in a single experiment would be highly problematic.

## Data Availability Statement

The binary of the model, running scripts and readme files can be found at https://github.com/JetBrains-Research/bcnnm-organoids/raw/master/public/fncom.2020.588224.zip. The source code of the model is provided at https://github.com/JetBrains-Research/bcnnm-organoids. The documentation of the model is provided at https://github.com/JetBrains-Research/bcnnm-organoids/tree/master/docs.

## Author Contributions

DB, GG, AP, SK, VM, and VS conceived the study. DB, GG, AP, and VM developed the framework. AP and VM wrote the analysis code. DB, GG, SK, and VS developed the configurations. All authors contributed to the writing and revising the manuscript. All authors read and approved the manuscript.

## Conflict of Interest

DB, GG, AP, VS, and SK were employed by legal entities which are a part of JetBrains group of companies. The remaining authors declare that the research was conducted in the absence of any commercial or financial relationships that could be construed as a potential conflict of interest.
