# Supplementary material for: BCNNM: A Framework for in silico Neural Tissue Development Modeling
Source: Front Comput Neurosci. 2021 Jan 20;14:588224. doi: 10.3389/fncom.2020.588224 (PMC7855713; doi:10.3389/fncom.2020.588224)
Supplement: Supplementary file 1 [file Data_Sheet_1.pdf]

Supplementary Material

1 SUPPLEMENTARY TABLES AND FIGURES

1.1 Figures

Figure S1

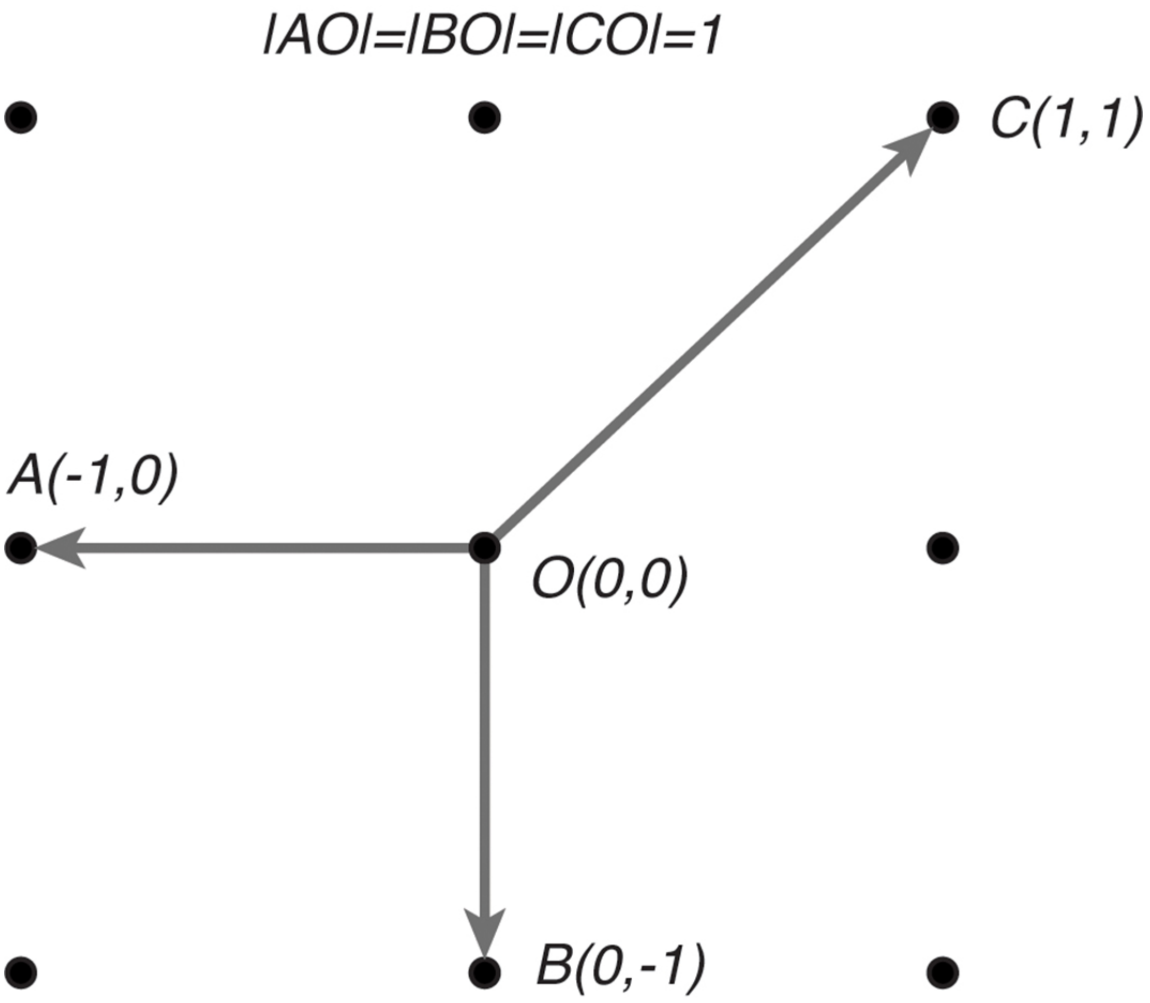

Figure S1. Minkowski space with Chebyshev metric. Our framework implements space in the form of an integer lattice with a limited number of equivalent displacements. In two-dimensional space, the number of possible displacements from the central coordinate O is $3^2 - 1$, in three-dimensional space is $3^3 - 1$.

Figure S2

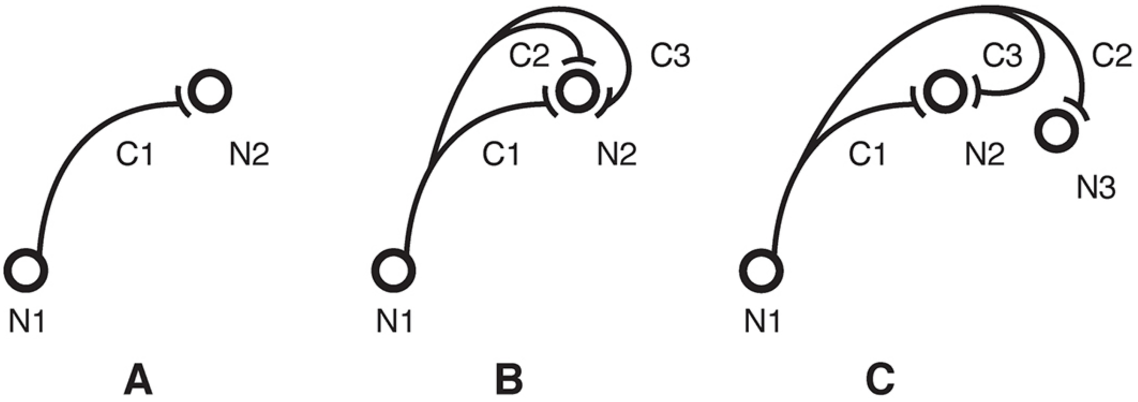

Figure S2. Single, sequentially repeated, non-sequentially repeated connections.

Figure S3

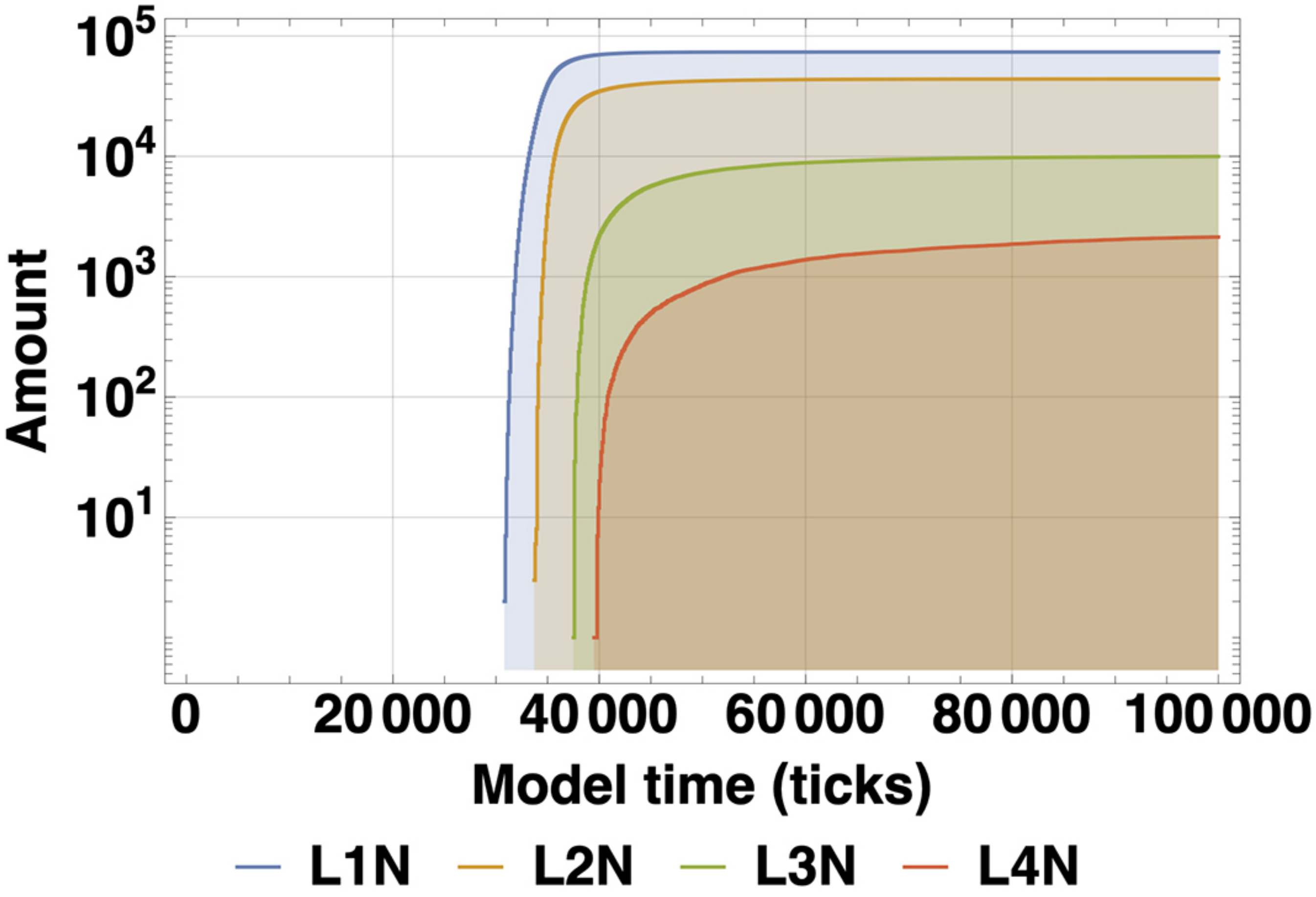

Figure S3. Neuron progenitors differentiation in time (Configuration II).

Figure S4

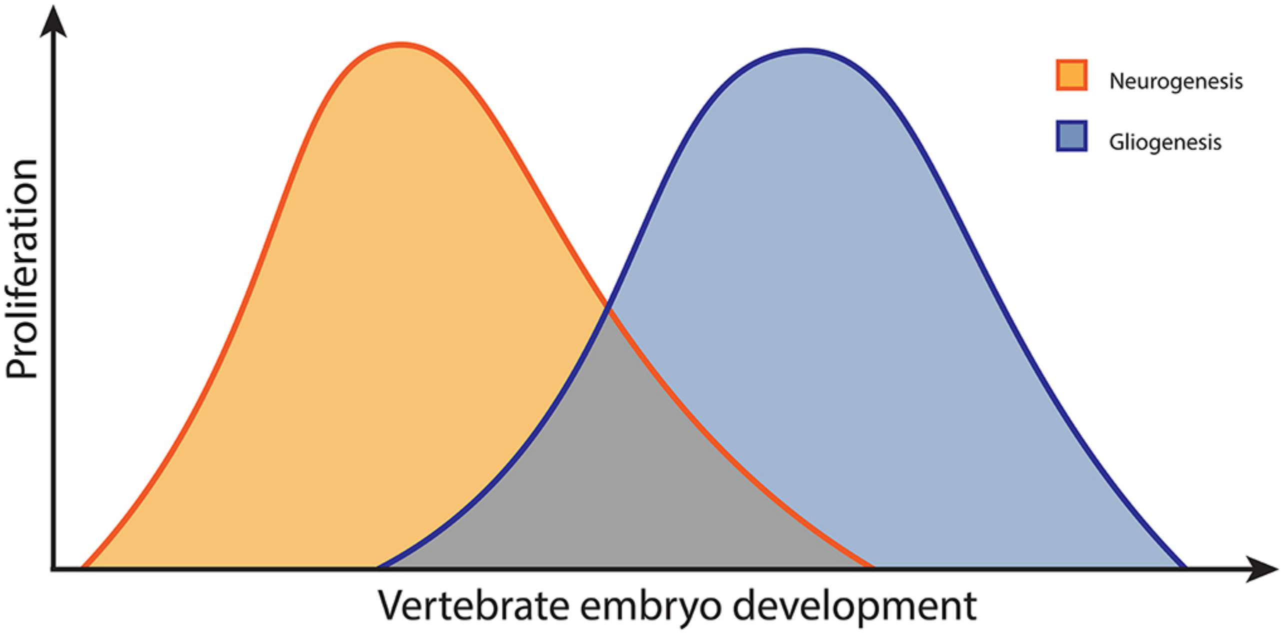

Figure S4. Temporal switch from neurogenic to gliogenic phase occurs in a developing vertebrate embryo. After Macht 2016, with changes.

Figure S5

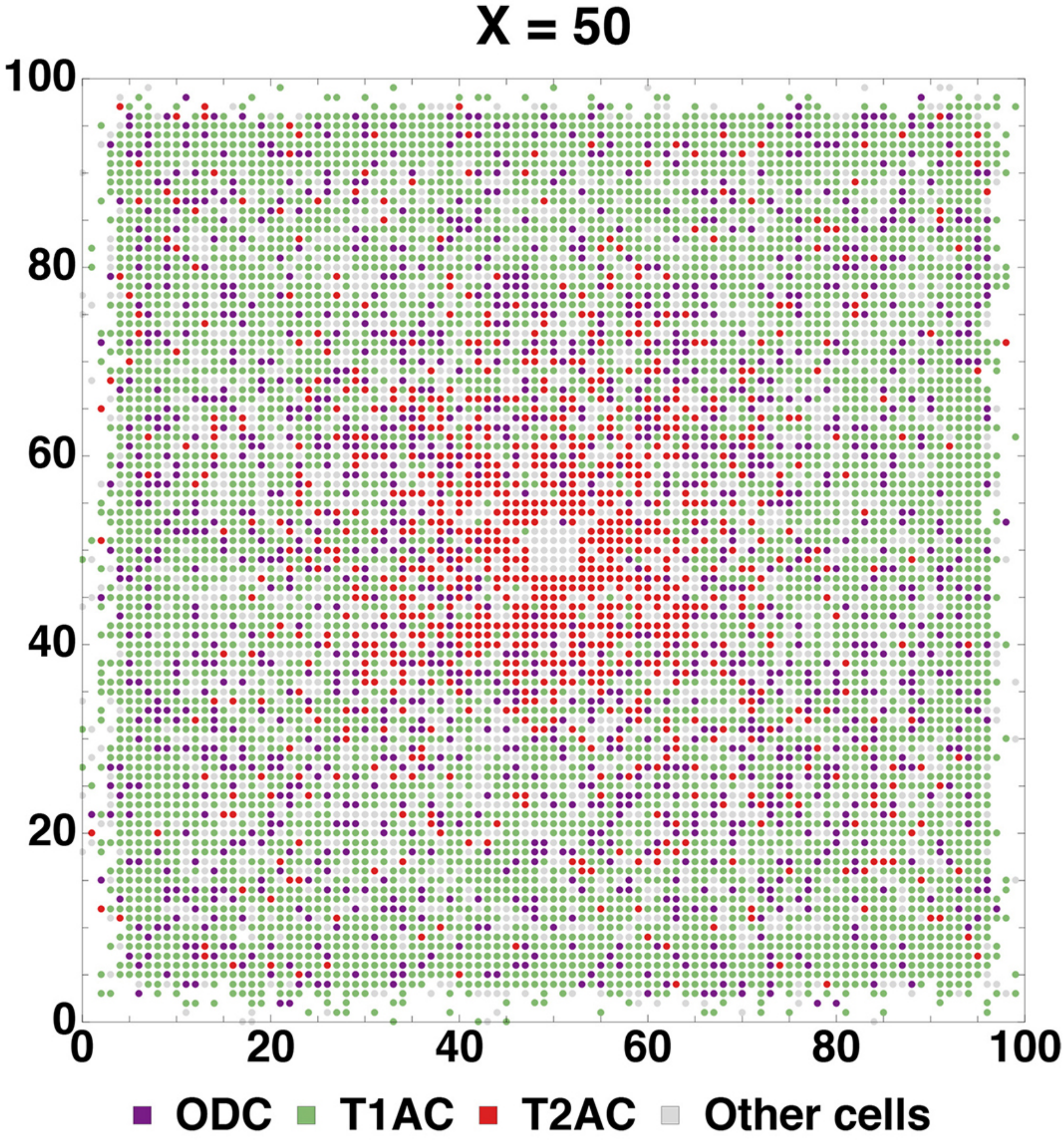

Figure S5. Cross-section of the resulting structure with glial cell types highlighted, based on Configuration II. Spatial arrangement of glial cell types differs depending on the distance from the symmetry center.

Figure S6

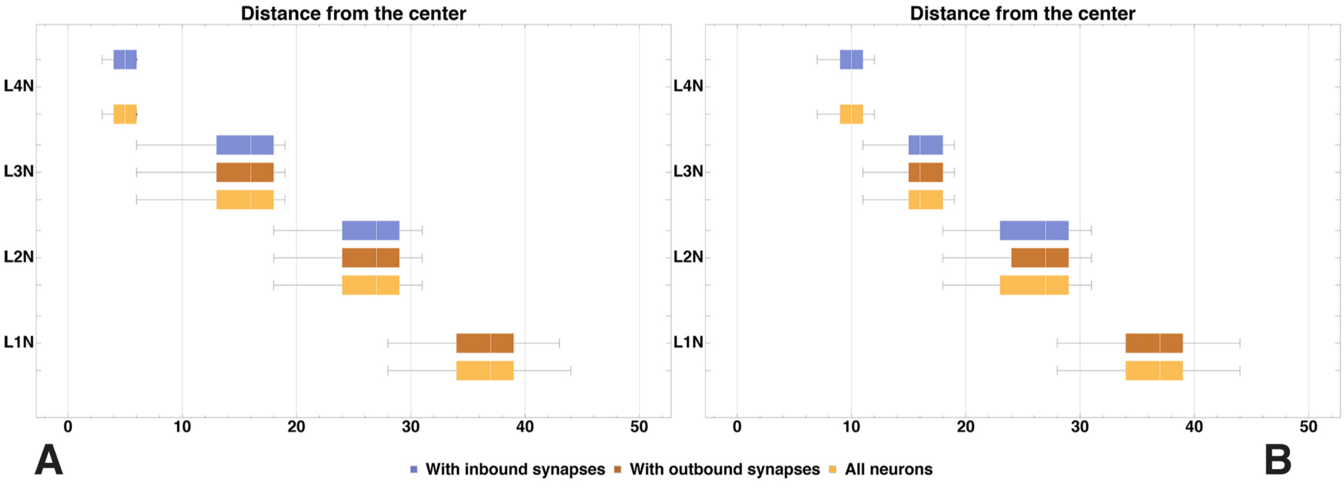

Figure S6. Spatial distribution of all neurons in comparison with neurons which have inbound and outbound synapses based on Configuration I (A) and Configuration II (B).

Figure S7

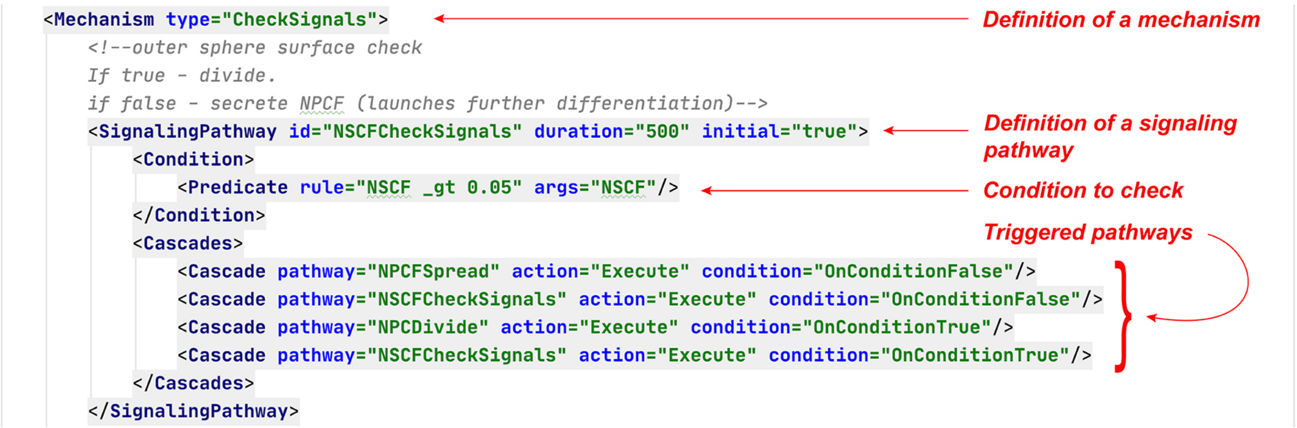

Figure S7. Example of XML configuration for mechanisms and conditions for Configuration II experiment.

Figure S8

```xml
<!--L3 Neuron with both dendrite and axon-->
<ModelCell baseType="NeuronCell" id="L3Neuron" immovable="true">
    <SPs>
        <SP type="NFSpread"/>
        <SP type="L3NeuronGNFCheckSignals"/>
        <SP type="L3NeuronAxonForm"/>
        <SP type="L3NeuronDendriteTreeForm"/>
    </SPs>
    <Compartments>
        <Compartment baseType="Axon" id="L3NeuronAxon" unique="true">
            <SPs>
                <SP type="L2NeuronAGFCheckSignal"/>
                <SP type="L3NeuronAxonGrowOn"/>
                <SP type="L3NeuronAxonGrowOff"/>
                <SP type="L3NeuronAxonalTerminalForm"/>
                <SP type="L2NeuronCAFCheckSignal"/>
            </SPs>
        </Compartment>
        <!--L3->l2-->
        <Compartment baseType="AxonalTerminal" id="L3NeuronAxonalTerminal" unique="false"...>

        <Compartment baseType="Synapse" id="L3NeuronSynapse" unique="false"/>

        <!--l1->L3-->
        <Compartment baseType="DendriteTree" id="L3NeuronDendriteTree" unique="true"...>

        <Compartment baseType="DendriticSpine" id="L3NeuronDendriticSpine" unique="false"...>
    </Compartments>
</ModelCell>
```

Definition of a cell type

Pathways available to the cell

Definition of a compartment

Pathways available to the compartment

Figure S8. Example of XML configuration for cell types for Configuration II experiment.

Figure S9

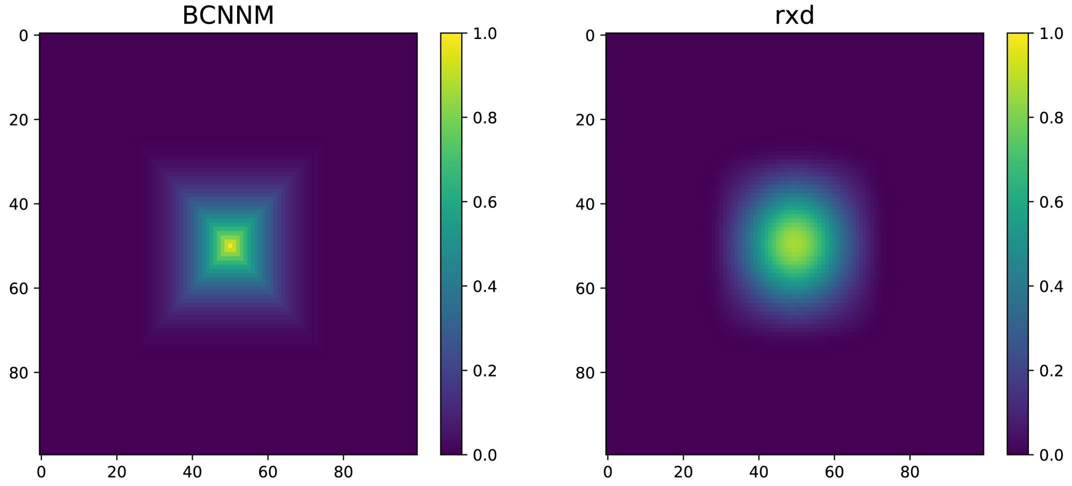

Figure S9. 2D representation of concentration gradients resulting from a single chemical factor diffusion in the BCNNM model (left) and simulated using the Gray-Scott equation (right).

Table 1

Comparison of performance results for Configuration I and II experiments.

Table S1. Statistics of simulations done on Intel Xeon processor 8 gen/64GB RAM.

Experiment	Model ticks	Simulation duration
Configuration I (A)	200 000	19m 26s
Configuration I (B)	3 000 000	4h 13m 00s
Configuration II (A)	200 000	18m 58s
Configuration II (B)	3 000 000	4h 12m 27s

2 SUPPLEMENTARY DATA

Data S1

Configuration files for all the experiments presented in this article, as well as the executable file of the model and quick start guide are available at: `https://github.com/JetBrains-Research/bcnnm-organoids/raw/master/public/fncom.2020.588224.zip`

Data S2

Events trace for cell with id = 1 in Configuration II experiment.

The data shows the sequence of events which displays transformations from Neural Progenitor Cell to Type 2 Astrocyte Cell and cell moving in model space.

∗Tick 1000: Neural Progenitor Cell (targetNPC) with id = 1 created as a result of asymmetrical division of starting Neural Stem Cell with id = 0. Coordinates of targetNPC is 49,50,51.

∗Tick 1600: Symmetrical division of targetNPC. Id of child is 2.

∗Tick 2100: Symmetrical division of targetNPC. Id of child is 4.

∗Tick 2600: Symmetrical division of targetNPC. Id of child is 6.

∗Tick 3100: Symmetrical division of targetNPC. Id of child is 12.

∗Tick 3600: Symmetrical division of targetNPC. Id of child is 22.

∗Tick 4100: Symmetrical division of targetNPC. Id of child is 44.

∗Tick 4600: Symmetrical division of targetNPC. Id of child is 85.

∗Tick 5100: Symmetrical division of targetNPC. Id of child is 165.

∗Tick 5400: Moving targetNPC to coordinate 48,51,50.

∗Tick 5600: Symmetrical division of targetNPC. Id of child is 312.

∗Tick 5800: Moving targetNPC to coordinate 47,51,49.

∗Tick 6100: Symmetrical division of targetNPC. Id of child is 583.

∗Tick 6600: Symmetrical division of targetNPC. Id of child is 1068.

∗Tick 6900: Moving targetNPC to coordinate 46,50,50.

∗Tick 7600: Symmetrical division of targetNPC. Id of child is 3237.

∗Tick 7600: Moving targetNPC to coordinate 45,51,49.

∗Tick 8000: Moving targetNPC to coordinate 44,50,49.

∗Tick 8600: Moving targetNPC to coordinate 43,49,48.

∗Tick 30700: Differentiation of targetNPC to RadialGliaCell. Id changed from 1 to 1849035.

∗Tick 40800: Differentiation from RadialGliaCell to GlialRestrictedProgenitorCell. Id changed from 1849035 to 3730866.

∗Tick 41600: Differentiation from GlialRestrictedProgenitorCell to OligodendrocyteType2AstrocytePrecursorCell. Id changed from 3730866 to 3765910.

∗Tick 42400: Differentiation from OligodendrocyteType2AstrocytePrecursorCell to Type2AstrocyteCell. Id changed from 3765910 to 3785489.
